# LINC01798/miR-17-5p axis regulates ITGA8 and causes changes in tumor microenvironment and stemness in lung adenocarcinoma

**DOI:** 10.3389/fimmu.2023.1096818

**Published:** 2023-02-23

**Authors:** Xuanguang Li, Guangsheng Zhu, Yongwen Li, Hua Huang, Chen Chen, Di Wu, Peijun Cao, Ruifeng Shi, Lianchun Su, Ruihao Zhang, Hongyu Liu, Jun Chen

**Affiliations:** ^1^ Department of Lung Cancer Surgery, Tianjin Medical University General Hospital, Tianjin, China; ^2^ Tianjin Key Laboratory of Lung Cancer Metastasis and Tumor Microenvironment, Tianjin Lung Cancer Institute, Tianjin Medical University General Hospital, Tianjin, China

**Keywords:** ITGA8, lncRNA, miRNA, LUAD, immune microenvironment, TMB, cancer cell stemness, regulatory network

## Abstract

Integrins are closely related to the occurrence and development of tumors. ITGA8 encodes the alpha 8 subunit of the heterodimeric integrin alpha8beta1. Studies on the role of this gene in the occurrence and development of lung cancer are scarce. The examination of public databases revealed that ITGA8 expression was significantly lower in tumor tissue than that in normal tissue, especially in lung cancer, renal carcinoma, and prostate cancer. Survival analysis of patients with lung adenocarcinoma revealed that higher ITGA8 expression had better prognosis. ITGA8 was positively related to immune checkpoints and immunomodulators, whereas B cell, CD4+ T cell, CD8+ T cell, neutrophil, macrophage, and dendritic cell infiltration had the same correlation. Moreover, ITGA8 was negatively related to cancer stemness. We used an online database to predict the miRNAs and lncRNAs that regulate ITGA8 and obtained the regulatory network of ITGA8 through correlation analysis and Kaplan–Meier survival analysis. Quantitative real-time PCR and western blot analyses showed that LINC01798 regulates ITGA8 expression through miR-17-5p. Therefore, the regulatory network of ITGA8 may serve as a new therapeutic target to improve the prognosis of patients with lung cancer.

## Introduction

Lung cancer is one of the most commonly occurring tumors in humans. In 2020, approximately 2.2 million new cases of lung cancer (accounting for 11.4% of the total incidence of cancer) and approximately 1.8 million deaths due to lung cancer (accounting for 18.0% of death due to cancer) were reported worldwide (1). However, effective therapies for lung cancer treatment are lacking (2), therefore, uncovering the mechanisms of lung cancer pathogenesis may address this problem.

Integrins are a major family of cell surface receptors, which are heterodimers composed of alpha and beta subunits. Integrins bind to the extracellular matrix and organize the cytoskeleton and activate intracellular signaling, regulating complex cellular behaviors, including survival, proliferation, and migration (3). Abnormal integrin expression is frequently associated with the occurrence and development of various diseases (4). The integrin family regulates various of steps cancer progression, such as tumor growth, metastasis, therapy resistance, and cancer stemness (5). Integrins have been implicated in acquired resistance to EGFR-TKIs in cancers of the lung (6), breast (7), and liver (8). Studies on the role of integrin in lung cancer mainly focused on integrin αvβ3, which can promote resistance to EGFR-TKIs through various mechanisms (9, 10). ITGA8 encodes the alpha 8 subunit of the heterodimeric integrin alpha8beta1, and data indicate that ITGA8 is closely related to tumorigenesis. For example, low ITGA8 expression is associated with poor prognosis in patients with clear cell renal cell carcinoma (11). High ITGA8 expression induces epithelial–mesenchymal transition in patients with early relapsed multiple myeloma and enhances cell migration and invasion (12). ITGA8 expression is related to the occurrence of colorectal cancer (13). However, the functional role of ITGA8 in lung cancer has not been characterized. In our study, initial exploration of the function of ITGA8 in lung cancer was performed through bioinformatics analysis.

## Materials and methods

### Dataset acquisition and processing

An interactive body map was obtained from Gene expression profiling interactive analysis (http://gepia.cancer-pku.cn). The RNA expression data, as well as the corresponding clinical data of lung adenocarcinoma (LUAD) and lung squamous cell carcinoma (LUSC) were downloaded from The Cancer Genome Atlas (TCGA) database (https://portal.gdc.cancer.gov/repository). The ITGA8 protein expression data of LUAD and LUSC were downloaded from the Human Protein Atlas (https://www.proteinatlas.org/).

### Evaluation of immune infiltration

We extracted the expression data of ITGA8, 150 marker genes of five types of immune pathways: chemokine (14), receptor (15), major histocompatibility complex [MHC, 21], immunoinhibitor (16), immunostimulator (17), and 60 marker genes of two types of immune checkpoint pathways: inhibitory (16) and stimulatory (18) in reference to studies in LUAD samples, and performed correlation analysis between them (19, 20).

Using the R software package ESTIMATE (21), we calculated the stromal, immune, and ESTIMATE scores in each tumor for each patient based on gene expression.

Using Timer (22) of the R software package IOBR (15), we reassessed B cell, CD4+ T cell, CD8+ T cell, neutrophil, macrophage, and dendritic cell (DC) infiltration scores in each tumor for each patient based on gene expression.

We obtained the cytotoxic T lymphocyte score of a LUAD dataset (GSE13213) with Tumor Immune Dysfunction and Exclusion (TIDE, http://tide.dfci.harvard.edu) to predict the response of patients to immunotherapy. We downloaded the scatter chart of correlation analysis between TIDE score (23) and ITGA8 gene expression on the official website of TIDE.

### Mutant Allele Tumor Heterogeneity Analysis

Somatic mutation data were extracted from the Genomic Data Commons database portal (https://portal.gdc.cancer.gov/), and the R software package Maftools (24) was used to calculate mutant allele tumor heterogeneity (MATH) and perform the visualization process. MATH can effectively represent the deviation of the distribution of mutation annotation format (MAF) values of tumor-specific sites, which indicates the degree to which MAF deviates from the overall MAF distribution of the sample. The larger the MATH value, the higher the tumor heterogeneity.

### Cancer cell stemness and gene expression

We obtained six cancer cell stemness scores calculated from mRNA expression (RNA expression-based stemness scores [RNAss], epigenetically regulated RNA expression-based stemness scores [EREG-EXPss]), DNA methylation signatures (DNA methylation-based stemness scores [DNAss], epigenetically regulated DNA methylation-based stemness scores [EREG-METHss], differentially methylated probes-based stemness scores [DMPss], and enhancer elements/DNA methylation-based stemness scores [ENHss]) from previous studies (25), and integrated the stemness scores and gene expression data of the samples for correlation analysis.

The role of ITGA8 in regulating cancer cell stemness was evaluated with Gene Oncology (GO) (26) and Kyoto Encyclopedia of Genes (KEGG) (27) enrichment analysis of the genes, which highly correlated with ITGA8 using Person’s correlation analysis (*r* > 0.7, *p* < 0.05), using “clusterProfiler” package in R.

### Construction of the ITGA8 regulatory network

The miRNAs that may affect ITGA8 expression were obtained through the online database The Encyclopedia of RNA Interactomes (ENCORI, http://starbase.sysu.edu.cn/), and the network map was created. Correlation analyses between the expression of these miRNAs and ITGA8 identified 3 miRNAs (*r* ≤ −0.2, *p* < 0.05). ENCORI identified 215 lncRNAs that could affect the expression of the 3 miRNAs. Correlation analyses between the expression of the 215 lncRNAs and ITGA8 in lung cancer identified 6 lncRNAs (*r* ≥ 0.4, *p* < 0.05) with high correlation. Finally, the regulatory network of ITGA8 was obtained.

### Cell culture and transient transfection

The human LUAD cell lines, A549, PC9, and H1975, and the normal human bronchial epithelial cell line, BEAS-2B, were obtained from the Cell Bank of the Chinese Academy of Sciences (Shanghai, China). The osimertinib (AZD9291)-resistant cell line H1975/AR was routinely cultured and screened in our laboratory after long-term stimulation with low-dose osimertinib. The abivertinib-resistant cell lines H1975/ABIR were routinely cultured and screened in our laboratory after long-term stimulation with low-dose abivertinib. All cell lines were cultured in RPMI 1640 medium containing 10% fetal bovine serum (Gibco, Carlsbad, CA) in the incubator at 37°C and humidified at 5% CO_2_ atmosphere. The small interfering RNA of LINC01798 and scramble siRNA (si-NC) were purchased from Ribobio (Guangzhou, China). The miR-17-5p inhibitor and miR-17-5p inhibitor NC were purchased from GenePharma (Shanghai, China). The miR-17-5p mimics and miR-17-5p mimics NC were purchased from Ribobio (Guangzhou, China). The overexpression vector of LINC01798 was constructed in pcDNA3.1 (+) by GENEWIZ (Suzhou, China). The cells were seeded in 6-well culture plates, and cultured overnight until 60%–70% confluence. Transient transfection was performed using Lipofectamine 3000 (Invitrogen, Carlsbad, CA) following the manufacturer’s instructions. Transfection efficiency was determined using quantitative real-time (qRT)-PCR and western blotting.

### Sphere formation assay

H1975 cells transfected with sh-NC and sh-ITGA8 (2000 cells/plate) were replated into 6-well ultra-low attachment culture dishes and cultured in RPMI 1640 containing 20 ng/mL b-FGF (Gibco), 20 ng/mL EGF (Gibco), 2% B27 supplement (Gibco), and 4 mg/mL insulin (Gibco). After 14 d, the spheroid colonies (>50 μm in diameter) were photographed and counted.

### RNA extraction, RT-PCR, and qRT-PCR

Total RNA was isolated using TRIzol Reagent (Invitrogen, Carlsbad, CA), and the quantity was measured by using NanoDrop equipment (Thermo Fisher Scientific, Waltham, MA, USA). Total RNA was reverse transcribed using PrimeScript RT reagent Kit (Takara) following the manufacturer’s instructions and qRT-PCR analyses were performed using qPCR SYBR Green Master Mix (Vazyme Biotechnology, Nanjing). All target genes were normalized to the endogenous reference gene β-actin by using the relative quantitative 2^−ΔΔCt^ method. U6 snRNA was used as the endogenous control for miR-17-5p.

### Western blot analysis

Cells were lysed using RIPA buffer (Beyotime Biotechnology, Shanghai, China) with 1 mM PMSF (Sigma, St Louis, USA) and phosphatase inhibitor (Beyotime Biotechnology, Shanghai, China) for 30 min on ice. Protein concentrations were determined and measured using a BCA assay (Thermo Fisher Scientific, Inc., Waltham, MA, United States). Equal amounts of protein (30 μg) were electrophoresed on 10% SDS-PAGE and transferred to the PVDF membrane (Millipore, Billerica, MA, United States). The membranes were blocked with 5% skim milk at 20-25 °C for 2 h. Subsequently, the membranes were incubated with primary antibody overnight at 4 °C under gentle rocking. Subsequently, the membranes and secondary antibody (1:5,000 dilution, Thermo Fisher Scientific, Inc.) were incubated for 1 h at 20-25 °C. The following were the antibodies used: GAPDH (1:2,000, ORIGENE) and ITGA8 (1:1,000, ABclonal).

### Human tissue and immunohistochemistry

Four non-small cell lung cancer (NSCLC) tissue samples were collected from patients between 2018 and 2020 at the Lung Cancer Surgery Department of Tianjin Medical University General Hospital. All participants provided written informed consent at the time of recruitment. All cases had pathologically confirmed diagnoses of NSCLC. Immunohistochemistry (IHC) was conducted in our study. The paraffin blocks were cut into 5-μm-thick slices, the tissue slices were first deparaffinized in xylene and then rehydrated with a gradient alcohol solution. After blocking with normal goat serum for 30 min, the sections were incubated with ITGA8-specific antibody (1:200 dilution) overnight at 4 °C. Subsequently, the sections were washed with PBS, and incubated with secondary antibody (ZSJQ, Beijing, China) for 30 min at 37 °C. Finally, the cells were incubated with 3,3′-diaminobenzidine for 2 min at 20-25 °C and counterstained with hematoxylin.

### Dual luciferase reporter assay

Use online target prediction tool (www.cuilab.cn/lnctar) to predict potential binding sites between miR-17-5p and LINC01798. The pmirGLO Dual-Luciferase miRNA Target Expression Vector was purchased from Promega. A wild-type (pmirGLO-LINC01798-WT) and mutant-type (pmirGLO-LINC01798-MUT) luciferase reporter gene plasmid containing the binding site were purchased from GENEWIZ (Suzhou, China). The cells (H1975) were seeded in 6-well culture plates, and cultured overnight until 70%–80% confluence. On the next day, the cells were co-transfected with pmirGLO-LINC01798-WT, pmirGLO-LINC01798-MUT reporter plasmids, and mimics NC, miR-17-5p mimics accordingly. 24 h post transfection, the luciferase activity was measured by GloMax 20/20 Luminometer (Promega) using the Dual-Luciferase Reporter Assay System (Beyotime Biotechnology, Shanghai, China) and normalized to Renilla luciferase activity respectively. Experiments were performed in triplicate.

### Statistical analysis

Statistical analysis was performed using the software GraphPad Prism 8 (GraphPad Software, La Jolla, CA) and SPSS version 23.0. Student’s t‐tests were used for the comparison of two sample groups. Mann–Whitney U and Kruskal–Wallis tests were applied to the examination of the relationship between ITGA8 expression and clinicopathological parameters. The relationship of ITGA8 with survival was studied using the Kaplan–Meier method, whereas significant differences between curves were evaluated using the log-rank test. The chi-square tests were used for the comparison of two groups of gene expression. P values < 0.05 were considered statistically significant.

## Results

### ITGA8 had lower expression in NSCLC

**Figure 1:**
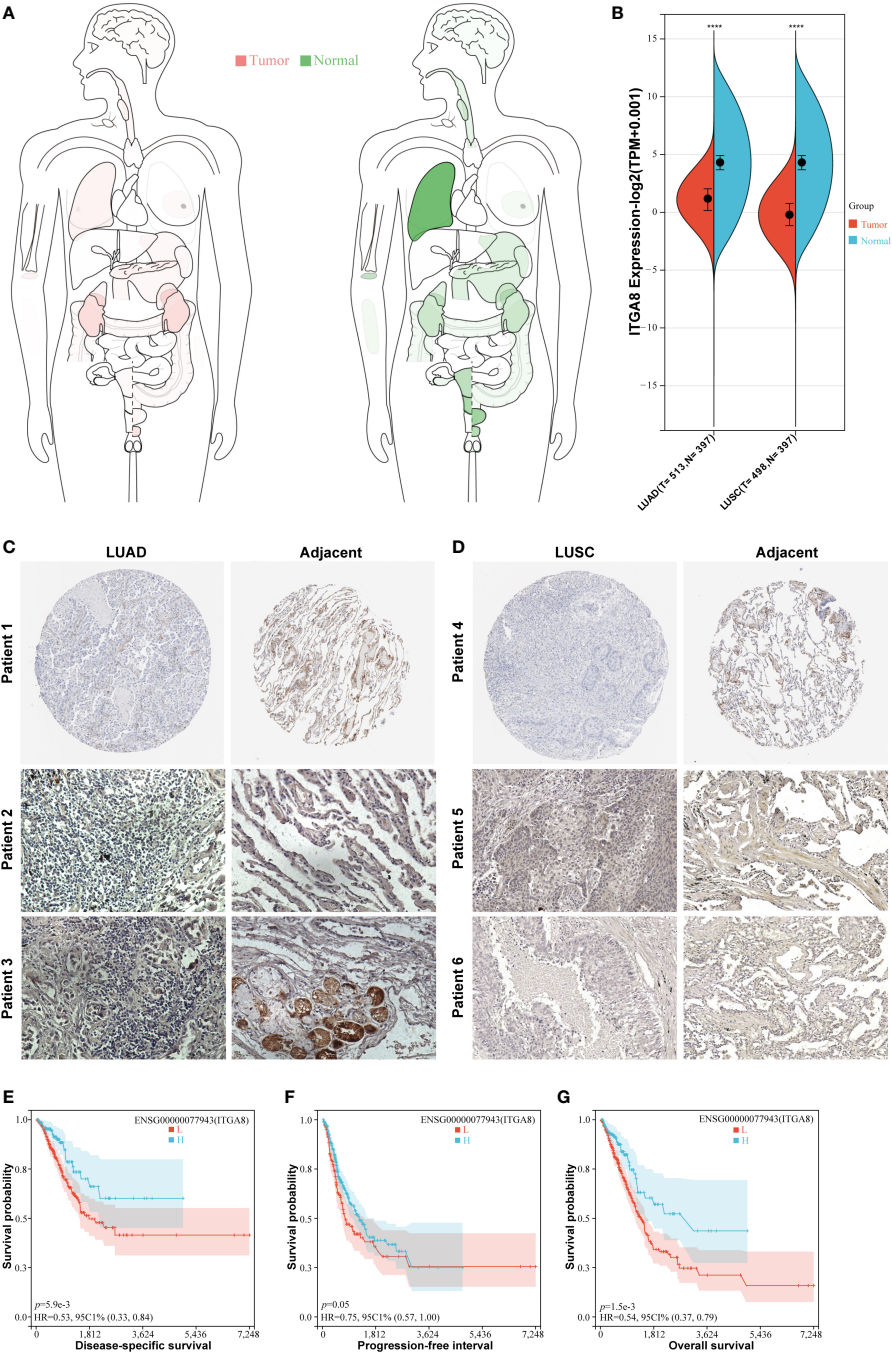
ITGA8 expression in non-small cell lung cancer (NSCLC) tissues and normal lung tissues and the prognostic value of ITGA8 in patients with LUAD. **(A)** ITGA8 gene expression data were acquired from TCGA and the body map of the median expression level of the ITGA8 gene in tumor and normal organs is shown. **(B)** The ITGA8 gene expression data were acquired from TCGA and the box diagram of ITGA8 expression in LUAD, LUSC, and matched normal lung tissues is shown. The expression level of ITGA8 gene was marked as Log 2(TPM+0.001) Scale. **(C, D)** The ITGA8 protein expression data of LUAD, LUSC, and matched normal lung tissues are shown. The data of patients 1 and 4 were downloaded from the Human Protein Atlas. The data of patients 2, 3, 5, and 6 were from our department. The patients with LUAD were divided into the high and low expression groups according to ITGA8 expression and Kaplan–Meier survival analysis was performed for **(E)** disease-specific survival (*p* = 0.0059), **(F)** progression-free interval (*p* = 0.05) and **(G)** overall survival (*p* = 0.0015). ****p < 0.0001.

To comprehensively evaluate the role of ITGA8 in multiple solid tumors, we used the TCGA dataset to examine ITGA8 expression in cancer. ITGA8 expression was significantly downregulated in lung, renal, rectal, and prostate cancers, suggesting that ITGA8 may be a tumor suppressor (**Figure 1A**). To examine the transcriptional expression of ITGA8 in NSCLC, we compared the expression of ITGA8 mRNA in normal lung and NSCLC tissues. The results showed that the expression of ITGA8 mRNA in LUAD and LUSC was lower than that in matched normal tissue (**Figure 1B**, *p* < 0.001). Consistently, the data in the Human Protein Atlas showed that the ITGA8 protein level was lower in LUAD (**Figure 1C**, patient 1) and LUSC (**Figure 1D**, patient 4) than that in normal tissues. The IHC results of patients with NSCLC in our department exhibited the same trend between LUAD and normal tissue (**Figure 1C**, patient 2, 3) and LUSC and normal tissue (**Figure 1D**, patient 5, 6), respectively.

### Patients with LUAD with high expression of ITGA8 mRNA had better prognoses

We analyzed the survival data in patients with LUAD. The group cutoff for high or low ITGA8 expression was set with a median. Patients with higher ITGA8 expression have longer DSS (Disease-Specific Survival, *p* = 0.0059) and PFI (Progression-Free Interval, *p* = 0.05) (**Figures 1E, F**). Furthermore, as the disease developed, patients with higher ITGA8 expression had a longer OS (Overall Survival, *p* = 0.0015, **Figure 1G**). In conclusion, ITGA8 may act as a good prognostic indicator for LUAD. Survival analysis results in patients with LUSC were not statistically significant (**Supplementary Figure 1**).

### High expression of ITGA8 mRNA in LUAD was associated with more immune infiltration

**Figure 2:**
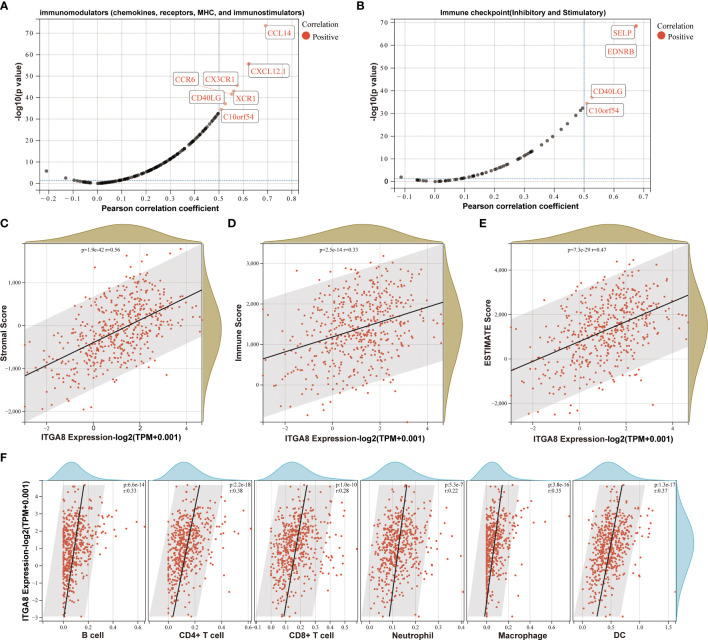
The relationship between ITGA8 and the immune microenvironment. **(A)** Correlation analysis between the expression of ITGA8 and 150 marker genes of five types of immune pathways in LUAD samples. **(B)** Correlation analysis between the expression of ITGA8 and 60 marker genes of two types of immune checkpoint pathways in LUAD samples. **(C)** Stromal (*r* = 0.56, *p* < 0.001), **(D)** immune (*r* = 0.33, *p* < 0.001), and **(E)** ESTIMATE scores (*r* = 0.47, *p* < 0.001) in each tumor for each patient based on gene expression level. **(F)** The infiltration scores of B cell (*r* = 0.33, *p* < 0.001), CD4+ T cell (*r* = 0.38, *p* < 0.001), CD8+ T cell (*r* = 0.28, *p* < 0.001), neutrophil (*r* = 0.22, *p* < 0.001), macrophage (*r* = 0.35, *p* < 0.001), and DC (*r* = 0.37, *p* < 0.001) in each tumor for each patient based on gene expression level. The expression level of ITGA8 gene was marked as Log 2(TPM+0.001) Scale.

Integrins play important roles in immune cells and the immune microenvironment (16). We found that chemokines (such as CCL14 and CXCL12.1), chemokines receptors (such as CCR6, CX3CR1, and XCR1), and immune checkpoints (such as SELP, EDNRB, CD40LG, and C10orf54) were positively correlated with the ITGA8 expression (**Figures 2A, B**). We separately calculated stromal (**Figure 2C**, *r* = 0.56, *p* < 0.001), immune (**Figure 2D**, *r* = 0.33, *p* < 0.001), and ESTIMATE scores (**Figure 2E**, *r* = 0.47, *p* < 0.001) in each tumor for each patient based on gene expression level. A positive correlation existed between ITGA8 expression and immune infiltration. We then reassessed the B cell, CD4+ T cell, CD8+ T cell, neutrophil, macrophage, and DC infiltration scores in each tumor for each patient based on gene expression level, and the results were all positively correlated with the ITGA8 expression and revealed more immune infiltration (**Figure 2F**, B cell, *r* = 0.33, *p* < 0.001; CD4+ T cell, *r* = 0.38, *p* < 0.001; CD8+ T cell, *r* = 0.28, *p* < 0.001; neutrophil, *r* = 0.22, *p* < 0.001; macrophage, *r* = 0.35, *p* < 0.001; and DC, *r* = 0.37, *p* < 0.001). Further, we assessed cytotoxic T lymphocyte score of LUAD based on ITGA8 expression, and the results showed that the score of cytotoxic T lymphocyte (CTL) was positively correlated with ITGA8 expression (**Supplementary Figure 2**), which revealed the higher ITGA8 expression, the better the efficacy of immunotherapy. The correlation between ITGA8 and immune infiltration revealed that the gene may play a significant role in immunotherapy.

### ITGA8 expression was negatively correlated with tumor mutational burden, neoantigen, and MATH

**Figure 3:**
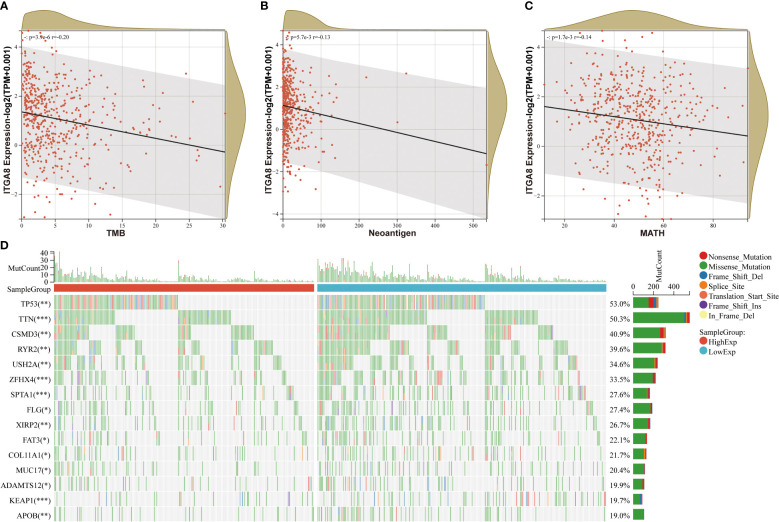
Correlation analyses between ITGA8 and TMB, neoantigen, and MATH. **(A)** Correlation analysis between the expression of ITGA8 and TMB in LUAD samples (*r* = −0.20, *p* < 0.001). **(B)** Correlation analysis between the expression of ITGA8 and neoantigen in LUAD samples (*r* = −0.13, *p* = 0.0057). **(C)** Correlation analysis between the expression of ITGA8 and MATH in LUAD samples (*r* = −0.14, *p* = 0.0017). **(D)** Mutation landscape map for the high and low expression groups of ITGA8 in patients with lung adenocarcinoma. *p < 0.05, **p < 0.01, ***p < 0.001. The expression level of ITGA8 gene was marked as Log 2(TPM+0.001) Scale.

We analyzed the correlation of ITGA8 and tumor mutational burden (TMB), neoantigen, and tumor heterogeneity to further explore the relationship between ITGA8 and immune microenvironment and immunotherapy. TMB, neoantigen, and tumor heterogeneity can be used to predict the response to immune checkpoint blockade (ICB) therapy and have emerged as useful biomarkers across multiple cancer types for identifying patients who would benefit from immunotherapy (28, 29). We conducted correlation analyses between ITGA8 and TMB, neoantigen, and MATH in LUAD, respectively. The results showed that TMB (**Figure 3A**, *r* = −0.20, *p* < 0.001), neoantigen (**Figure 3B**, *r* = −0.13, *p* = 0.0057), and MATH (**Figure 3C**, *r* = −0.14, *p* = 0.0017) were negatively correlated with ITGA8 expression. Subsequently, we created a mutation landscape map for the high and low expression groups of ITGA8 in patients with LUAD and used the chi-square test to evaluate the difference in gene mutation frequency in each group of samples. The following were the top five genes with mutations: TP53, TIN, CSMD3, RYR2, and USH2A (**Figure 3D**).

### ITGA8 expression was negatively correlated with cancer cell stemness

**Figure 4:**
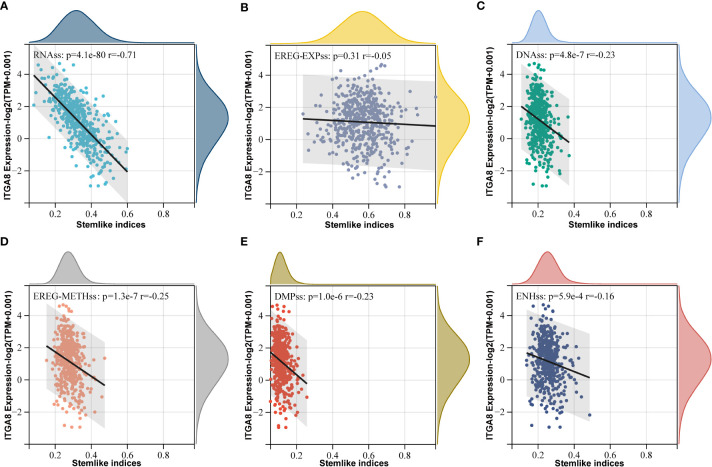
ITGA8 and cancer cell stemness. Correlation analyses between six tumor stemness scores and the gene expression data of ITGA8. **(A)** RNAss (*r* = −0.71, *p* < 0.001). **(B)** EREG-EXPss (*r* = −0.05, *p* = 0.31). **(C)** DNAss (*r* = −0.23, *p* < 0.001). **(D)** EREG-METHss (*r* = −0.25, *p* < 0.001). **(E)** DMPss (*r* = −0.23, *p* < 0.001). **(F)** ENHss (*r* = −0.16, *p* < 0.001). The expression level of ITGA8 gene was marked as Log 2(TPM+0.001) Scale.

ITGA8 was closely related to cancer stem cell (CSC), which plays an important role in immune evasion (3). Some studies have shown that cancer cell stemness affects the efficacy of immunotherapy for various tumors (30–32). We calculated cancer cell stemness scores and performed correlation analyses to investigate the relationship between ITGA8 and cancer cell stemness. The cancer cell stemness score is a measure of the similarity of tumor cells to stem cells, which is associated with active biological processes in stem cells and a higher degree of tumor dedifferentiation. All six cancer cell stemness scores were negatively correlated with ITGA8 expression (**Figure 4A**, RNAss, *r* = −0.71, *p* < 0.001; **Figure 4B**, EREG-EXPss, *r* = −0.05, *p* = 0.31; **Figure 4C**, DNAss, *r* = −0.23, *p* < 0.001; **Figure 4D**, EREG-METHss, *r* = −0.25, *p* < 0.001; **Figure 4E**, DMPss, *r* = −0.23, *p* < 0.001; **Figure 4F**, ENHss, *r* = −0.16, *p* < 0.001). We conducted a sphere formation assay and western blot to verify the results. The sphere formation assay revealed that knockdown of ITGA8 enhanced sphere-forming abilities (**Supplementary Figures 3A, B**). The results of western blot analysis showed that ITGA8 negatively regulated the expression of stemness markers, whereas these proteins were upregulated in ITGA8-knockdown cells (**Supplementary Figure 3C**). Collectively, these results demonstrated that high ITGA8 expression may contribute to good immunotherapy efficacy.

Furthermore, we conducted GO, and KEGG enrichment analyses for genes, which were highly correlated with ITGA8 through Person’s correlation analysis (*r* > 0.7, *p* < 0.05), and the most common significantly altered pathways in LUAD were focal adhesion and cell adhesion (**Supplementary Figures 4A, B**). Integrins are tightly involved in cell-extracellular matrix adhesion and extracellular chemical/biomechanical signal transduction, which regulates the activation of anti-apoptosis and pro-survival signaling pathways by interacting with matrix elements, such as collagen and fibrin (33, 34). Cell adhesion molecules, such as CD44v isoforms, and CD146 (35, 36) and focal adhesion molecules, such as FAK (37, 38), play essential roles in cancer cell stemness. Therefore, we can speculate that ITGA8 affects cancer cell stemness through the cell adhesion and focal adhesion pathways.

### LINC01798 regulates ITGA8 expression through miR17-5p in LUAD

**Figure 5:**
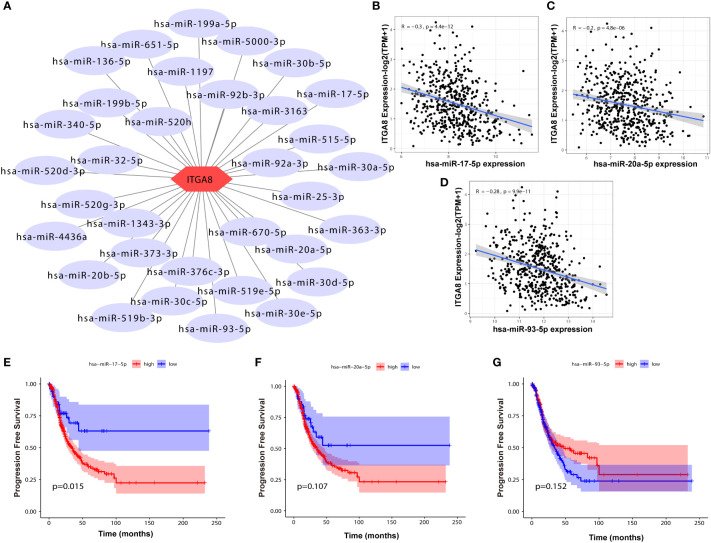
Correlation analyses between the expression of miRNAs and ITGA8. **(A)** The network map of miRNAs that may affect the expression of ITGA8. **(B)** Correlation analysis between the expression of miR-17-5p and ITGA8 (*r* = −0.3, *p* < 0.001). **(C)** Correlation analysis between the expression of miR-20a-5p and ITGA8 (*r* = −0.2, *p* < 0.001). **(D)** Correlation analysis between the expression of miR-93-5p and ITGA8 (*r* = −0.28, *p* < 0.001). PFS-based Kaplan–Meier survival analyses of **(E)** miR-17-5p (*p* = 0.015), **(F)** miR-20a-5p (*p* = 0.107), and **(G)** miR-93-5p (*p* = 0.152). The expression level of ITGA8 gene was marked as Log 2(TPM+1) Scale.

We determined regulatory genes upstream through an online database to explore the regulatory role of ITGA8 in lung cancer. The miRNAs that may affect ITGA8 expression were obtained through the online database, and the network map was created (**Figure 5A**). We performed correlation analyses between the expression of these miRNAs and ITGA8 in lung cancer, and 3 miRNAs (*r* ≤ −0.2, *p* < 0.05) were screened out (**Figure 5B**, miR-17-5p, *r* = −0.3, *p* < 0.001; **Figure 5C**, miR-20a-5p, *r* = −0.2, *p* < 0.001; **Figure 5D**, miR-93-5p, *r* = −0.28, *p* < 0.001). PFS-based Kaplan–Meier survival analyses (**Figure 5E**, miR-17-5p, *p* = 0.015; **Figure 5F**, miR-20a-5p, *p* = 0.107; **Figure 5G**, miR-93-5p, *p* = 0.152) and OS-based Kaplan–Meier survival analyses were performed on the 3 miRNAs (**Supplementary Figure 5**).

**Figure 6:**
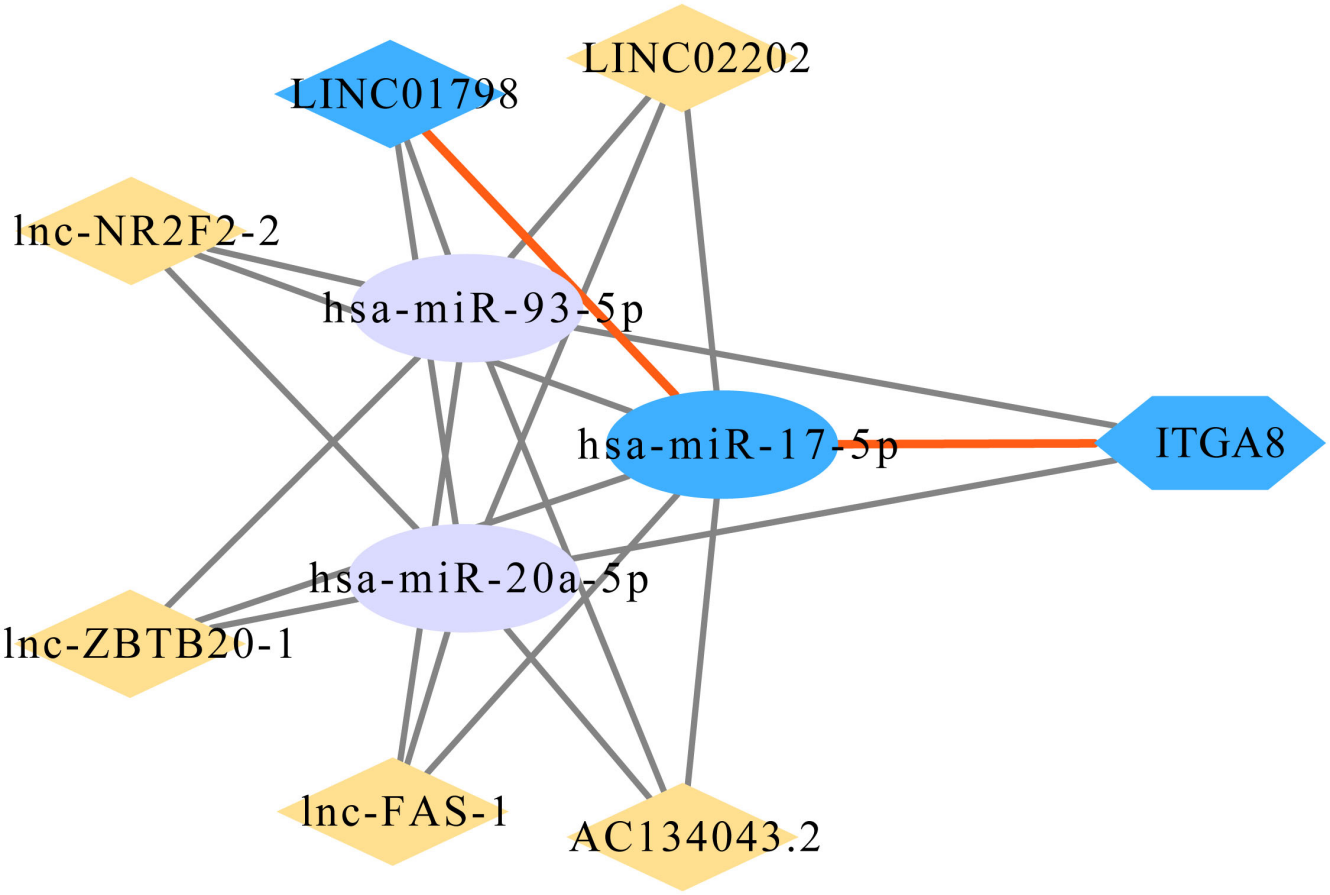
Gene regulatory network of ITGA8.

A total of 215 lncRNAs that might affect the expression of the 3 miRNAs were obtained through the online database, and correlation analyses were performed between the expression of these lncRNAs and ITGA8 in lung cancer. Six lncRNAs (*r* ≥ 0.4, *p* < 0.05) with high correlation were screened out (**Supplementary Figures 6A–F**), and Kaplan–Meier survival analyses were performed (**Supplementary Figures 7A–L**). Finally, the ITGA8 regulatory network was obtained (**Figure 6**). According to the results of the Kaplan–Meier survival analysis, we selected LINC01798 (PFS, *p* = 0.0074; OS, *p* = 0.00024) and has-miR-17-5p (PFS, *p* = 0.015; OS, *p* = 0.102) for experimental verification.

**Figure 7:**
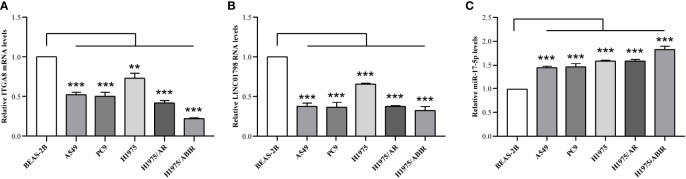
LINC01798, miR-17-5p, and ITGA8 expression in 5 LUAD cell lines and 1 human bronchial epithelial cell line as determined by qRT-PCR. Expressions of **(A)** ITGA8, **(B)** LINC01798, and **(C)** miR-17-5p in LUAD cell lines (A549, PC9, H1975, H1975/AR, and H1975/ABIR) and a human bronchial epithelial cell line (BEAS-2B) are analyzed by qRT-PCR. **p < 0.01, ***p < 0.001.

**Figure 8:**
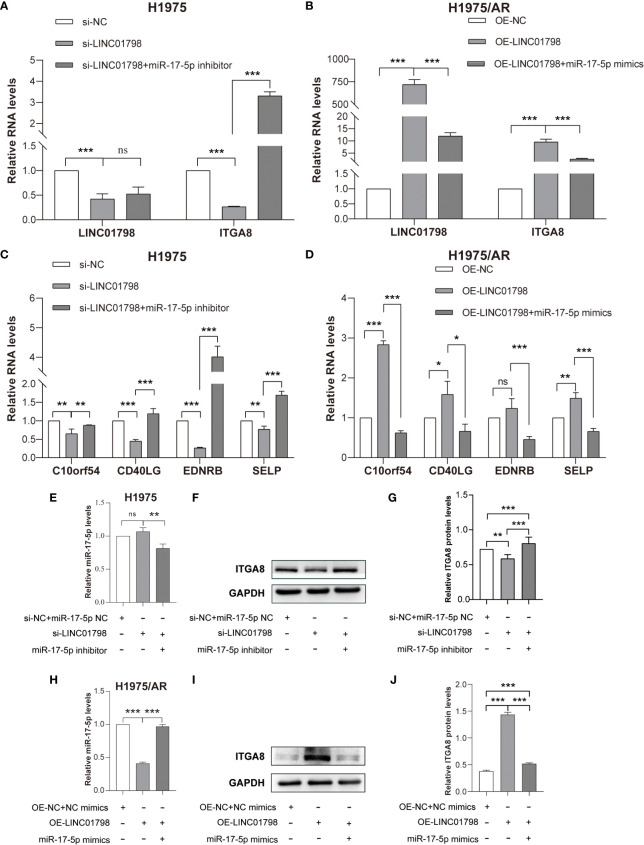
Reciprocal correlation among LINC01798, miR-17-5p, and ITGA8. **(A)** Relative RNA levels of LINC01798 and ITGA8 in H1975 after being transfected with si-NC + miR-17-5p inhibitor NC, si-LINC01798, and si-LINC01798 + miR-17-5p inhibitor. **(B)** Relative RNA levels of LINC01798 and ITGA8 in H1975/AR after being transfected with pcDNA3.1-NC + NC mimics, pcDNA3.1-LINC01798, and pcDNA3.1-LINC01798 + miR-17-5p mimics. **(C)** Relative mRNA levels of immune checkpoint genes in H1975 after being transfected with si-NC + miR-17-5p inhibitor NC, si-LINC01798, and si-LINC01798 + miR-17-5p inhibitor. **(D)** Relative mRNA levels of immune checkpoint genes in H1975/AR after being transfected with pcDNA3.1-NC + NC mimics, pcDNA3.1-LINC01798, and pcDNA3.1-LINC01798 + miR-17-5p mimics. **(E)** Relative miR-17-5p level in H1975 after being transfected with si-NC + miR-17-5p inhibitor NC, si-LINC01798, and si-LINC01798 + miR-17-5p inhibitor. **(F, G)** Relative protein level of ITGA8 in H1975 after being transfected with si-NC + miR-17-5p inhibitor NC, si-LINC01798, and si-LINC01798 + miR-17-5p inhibitor. **(H)** Relative miR-17-5p level in H1975/AR after being transfected with pcDNA3.1-NC + NC mimics, pcDNA3.1-LINC01798, and pcDNA3.1-LINC01798 + miR-17-5p mimics. **(I, J)** Relative protein level of ITGA8 in H1975/AR after being transfected with pcDNA3.1-NC + NC mimics, pcDNA3.1-LINC01798, and pcDNA3.1-LINC01798 + miR-17-5p mimics. *p < 0.05, **p < 0.01, ***p < 0.001, ns (not statistically significant) *p* > 0.05.

ITGA8 expression is lower in LUAD than in normal lung tissue (**Figures 1B, C**). To explore the aberrant expression of LINC01798, miR-17-5p, and ITGA8 at cellular levels, we selected human LUAD cell lines, A549, PC9, H1975, H1975/AR, and H1975/ABIR, and the human bronchial epithelial cell line BEAS-2B. The qRT-PCR assay revealed that the ITGA8 expression and LINC01798 expression in LUAD cell lines were significantly lower than that in normal lung tissue cell line (**Figures 7A, B**). By contrast, the miR-17-5p expression in LUAD cell lines was significantly higher than that in normal lung tissue cell line (**Figure 7C**).

si-LINC01798 was transfected into H1975 to investigate the effects of LINC01798 on LUAD cell function. The results showed that the expression of LINC01798 was knocked down, and ITGA8 expression was downregulated at mRNA and protein levels (**Figures 8A, F, G**), whereas the expression of miR-17-5p was slightly upregulated without statistical significance (**Figure 8E**). When further transfected with the si-LINC01798 and miR-17-5p inhibitor, the downregulation of ITGA8 was reversed, even higher than that in the control group (**Figures 8A, F, G**). When LINC01798 was knocked down, the immune checkpoint genes, SELP, EDNRB, CD40LG, and C10orf54, which were positively correlated with ITGA8 expression, were reduced at the mRNA level, and miR-17-5p inhibitor could reverse their reduction (**Figure 8C**). We constructed a LINC01798 overexpression vector using pcDNA3.1. LINC01798 expression level was significantly elevated after transfection of pcDNA3.1-LINC01798 in H1975/AR cells (**Figure 8B**). ITGA8 expression was upregulated at mRNA and protein levels (**Figures 8B, I, J**), whereas the miR-17-5p expression was downregulated (**Figure 8H**). When further transfected with the pcDNA3.1-LINC01798 and miR-17-5p mimic, the upregulation of ITGA8 and LINC01798 was reversed (**Figures 8B, I, J**). When LINC01798 was overexpressed, the immune checkpoint genes were elevated at the mRNA level, and miR-17-5p mimic could reverse this increase (**Figure 8D**). The transfection of miR-17-5p mimic decreased LINC01798 expression in H1975/AR cells (**Figure 8B**), and conversely, transfection of miR-17-5p inhibitor slightly increased LINC01798 expression in H1975 cells. The overexpression of LINC01798 decreased miR-17-5p expression in H1975/AR cells (**Figure 8H**) and transfection of si-LINC01798 slightly increased miR-17-5p expression (**Figure 8E**).

**Figure 9:**
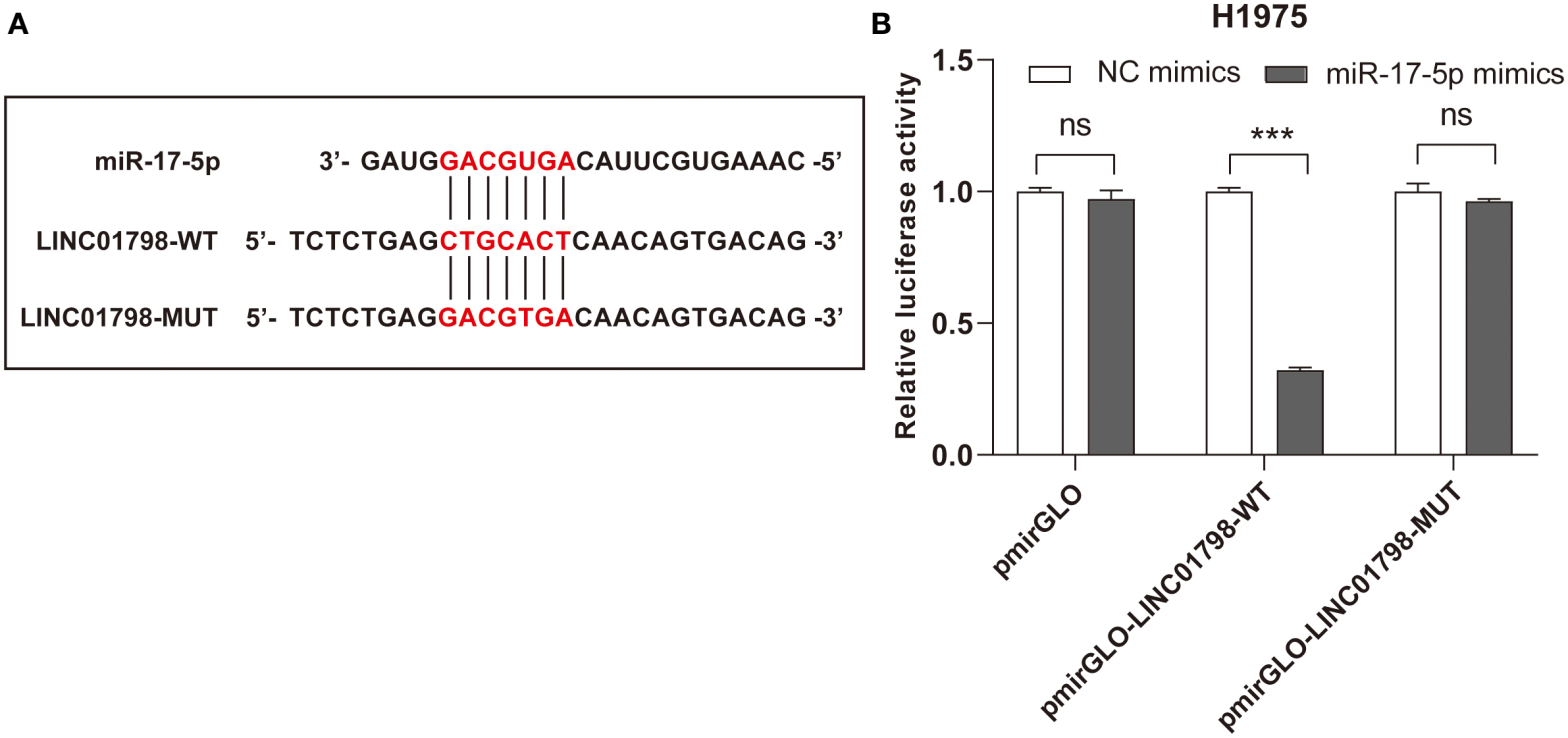
Reciprocal correlation between LINC01798 and miR-17-5p through the dual luciferase reporter assay. **(A)** The potential binding sites of miR-17-5p to the LINC01798 sequence. **(B)** The results of the dual luciferase reporter gene assay. ***p < 0.001, ns (not statistically significant) *p* > 0.05.

The potential binding sites between LINC01798 and miR-17-5p are shown in **Figure 9A**. Compared with the control group, cotransfection with pmirGLO-LINC01798-WT vector and miR-17-5p mimic reduced luciferase reporter activity significantly in H1975 cells (**Figure 9B**). This repressive effect was abrogated by mutations of the miR-17-5p binding region in LINC01798 (**Figure 9B**). In conclusion, LINC01798 regulated ITGA8 expression indirectly by sponging miR-17-5p.

## Discussion

Integrins, which mediate cell adhesion and transmit mechanical and chemical signals to the interior of cells, are closely related to the occurrence and development of tumors. Uncontrolled integrin signaling in cancer allows tumor cells to proliferate unrestrictedly, invade tissue boundaries, and increase tumor cell viability in ectopic microenvironments (3). Integrins control all aspects of cancer development not only by supporting mitosis and survival signaling, but also more directly, from tumor initiation and initial invasion to metastatic reactivation of dormant disseminated tumor cells (18, 39). Positional control of the actions of cytokine and growth factor receptors to coordinate development, regeneration, and various repair processes is the most important function of integrins (40). For example, integrins and receptor tyrosine kinases (RTKs) work together to ensure optimal activation of promitogenic and prosurvival signaling through Ras-extracellular signal-regulated kinase (ERK) and phosphatidylinositol 3-kinase (PI3K)-AKT signaling pathways. To support anchorage-dependent cell survival and proliferation, the signaling pathways activated by integrins and RTKs are extensively interconnected at numerous levels (3). The tight association of integrins with RTKs may also play a significant role in the acquired resistance of EGFR-TKIs. ITGA8 encodes the α8 subunit of integrin α8β1. Studies on the relationship between ITGA8 and lung cancer are scarce and therefore, warranted.

In this study, we conducted a comprehensive bioinformatics analysis of public sequencing data and found that ITGA8 expression in LUAD and LUSC was lower than that in normal tissues. Data in Human Protein Atlas and the results of IHC showed the same results at the protein level. ITGA8 expression was significantly lower in LUAD cell lines than in human bronchial epithelial cell lines. Additionally, Kaplan–Meier survival analysis showed that in LUAD, patients with high ITGA8 expression were positively correlated with DSS, PFI, and OS, indicating that ITGA8 might be a tumor suppressor, and was positively correlated with good prognosis in LUAD. Therefore, ITGA8 may be used as a diagnostic, therapeutic, and prognostic biomarker for LUAD.

Integrins play important roles in immune cells and the immune microenvironment. For example, integrin αvβ6 is an important factor in the recruitment and retention of DCs and T cells in epithelial cells of tissues such as the gut, skin, and lung and may promote T cell recruitment to certain tumors (41). Integrin-dependent TGF-β activation in the immune system, which regulates T cell population generation and maintenance, antibody response, and pathogen clearance, plays a key role in autoimmune and inflammatory diseases (14, 16, 42). In this study, we analyzed the expression data of ITGA8 and five types of immune pathway marker genes and two types of immune checkpoint pathway marker genes in LUAD and found that ITGA8 expression was positively correlated with the expression of these genes, whereas the ITGA8 expression was also positively correlated with the immune infiltration scores. These results suggest that ITGA8 is inextricably linked to the immune microenvironment and may be used as a biomarker to predict or evaluate the efficacy of immunotherapy.

Many studies have revealed that patients with high TMB consistently benefit from ICB therapy, and the current TMB threshold for predicting the benefit from ICB therapy was approximately 200 non-synonymous somatic mutations by whole exome sequencing in lung, bladder, head, and neck cancers. In addition, the PD-L1 expression affected the response of single-agent PD-L1 antibody to ICB in tumors with high TMB (28). Although some studies have shown that high TMB might not accurately predict the ICB response of all cancer types (43), TMB is currently the most widely used biomarker for evaluating immunotherapy efficacy. Most clinical studies using TMB as a biomarker to predict the efficacy of immunotherapy have been successfully completed. Furthermore, a few somatic mutations in tumor DNA can generate neoantigens, and mutation-derived antigens are recognized and targeted by the immune system, especially after treatment with T cell-activating drugs (44, 45). These mutations can be transcribed and translated, and the neoantigen-containing peptides can be processed by antigen-processing machinery and loaded onto MHC molecules for presentation on the cell surface, where they can be recognized by T cells (46). The more somatic mutations a tumor has, the more neoantigens it may also form (43). In our study, ITGA8 expression is negatively correlated with TMB and neoantigen, suggesting that immunotherapy may be poor in patients with high ITGA8 expression. This may be related to the separate analysis of the correlation between TMB/neoantigen and ITGA8 without the expression of PD-L1. Moreover, tumor heterogeneity is closely related to immunotherapy. Some studies have shown that tumor heterogeneity was paramount for tumors to emerge as they eluded natural immune surveillance, progress, and resist immunotherapy (17, 47). In addition, high genetic and nongenetic tumor heterogeneity have been associated with poor patient outcomes (48, 49). We performed a correlation analysis between ITGA8 and MATH in LUAD. The results showed a negative correlation, revealing that patients with high ITGA8 expression had low tumor heterogeneity and good prognoses. In the mutation landscape map of high and low ITGA8 expression groups in patients with LUAD, the top five genes with mutations were TP53, TIN, CSMD3, RYR2, and USH2A. If ITGA8 is used as a biomarker for lung cancer diagnosis, treatment, and prognosis in the future, the expression of these genes may support it.

CSCs play an important role in tumor development, recurrence, and metastasis, and the intrinsic self-renewal and tumorigenic properties of these cells provide them with a unique ability to resist various forms of anticancer therapy, seed recurrent tumors, and disseminate and colonize to distant tissues (50). In glioblastoma multiforme (GBM) and skin tumors, CSCs promote their expansion through niche–integrin signals, and the production of the proangiogenic cytokine, vascular endothelial growth factor (VEGF), induces perivascular niche formation for CSC expansion. In addition to inducing neovascularization, VEGF also binds to VEGF receptor 2 (VEGFR2) and its coreceptor neuropilin 1 on CSCs to stimulate their self-renewal in an autocrine manner (3, 51). In triple-negative breast cancer, α6β1 integrin binds to the VEGFR2–neuropilin 2 complex to promote FAK and ERK activation, thereby promoting the transcription of hedgehog signaling (52, 53). Mechanistic studies of CSCs resistant to EGFR-TKIs have linked the ability of αvβ3 to promote stemness to activation of KRAS and signaling to NF-κB (54). These findings suggest that multiple integrin signaling mechanisms promote the self-renewal and expansion capabilities of CSCs in a context-dependent manner; whether CSCs are more dependent on integrin signaling than normal stem cells or rely on specific integrin signaling pathways or mechanisms that can be safely targeted for therapy remains to be determined. In our study, the six stemness scores were negatively correlated with ITGA8 expression. We can presume that the patients with high ITGA8 expression have lower stemness scores in the tumor, good immunotherapy efficacy, and good prognoses.

Finally, through the online database, we mapped the gene regulatory network of ITGA8, and we selected LINC01798/miR-17-5p/ITGA8 for experimental validation through the results of survival analysis. The expression of LINC01798 and ITGA8 in LUAD cell lines was significantly lower than that in the normal lung tissue cell line, whereas the expression of miR-17-5p was higher than that in the normal lung tissue cell line. When LINC01798 was knocked down, the expression of ITGA8 was significantly downregulated at the mRNA and protein levels, and the miR-17-5p expression was slightly increased. We can presume that when LINC01798 is knocked down, its capacity to sponge miR-17-5p is decreased; therefore, the inhibition of miR-17-5p to ITGA8 is enhanced. The miR-17-5p inhibitor could reverse the process. Moreover, when LINC01798 was knocked down, the immune checkpoint genes, SELP, EDNRB, CD40LG, and C10orf54, which were positively correlated with ITGA8 expression, were downregulated at the mRNA level. Moreover, miR-17-5p inhibitor could reverse their reduction. Conversely, similar correlations were detected when LINC01798 was overexpressed and miR-17-5p mimic could reverse the process. Therefore, the regulatory network of ITGA8 is feasible.

In conclusion, ITGA8 expression is downregulated in LUAD, and patients with high ITGA8 expression indicate good prognoses. Furthermore, ITGA8 is closely related to the immune microenvironment, TMB, tumor heterogeneity and cancer cell stemness. ITGA8 may be used as a diagnostic, therapeutic, and prognostic biomarker for LUAD in the future. Finally, the gene regulatory network of ITGA8 constructed through bioinformatics analysis, can help to explore the molecular mechanism of lung cancer and discover novel therapeutic targets.

## Data availability statement

The original contributions presented in the study are included in the article/**Supplementary Material**. Further inquiries can be directed to the corresponding authors.

## Ethics statement

The studies involving human participants were reviewed and approved by Tianjin Medical University General Hospital. The patients/participants provided their written informed consent to participate in this study.

## Author contributions

JC and HL contributed to the study conception and design. XL, GZ, and YL conducted the data analysis. HH, DW, and RS performed the data collection. PC, CC, LS, and RZ conducted material preparation. The first draft of the manuscript was written by XL and all authors commented on previous versions of the manuscript. All authors contributed to the article and approved the submitted version.
