# Supplementary material for: LINC01798/miR-17-5p axis regulates ITGA8 and causes changes in tumor microenvironment and stemness in lung adenocarcinoma
Source: Front Immunol. 2023 Feb 23;14:1096818. doi: 10.3389/fimmu.2023.1096818 (PMC9995370; doi:10.3389/fimmu.2023.1096818)
Supplement: Supplementary file 1 [file DataSheet_1.docx]

Supplementary Material

# Supplementary Figures and Tables

## Supplementary Figures


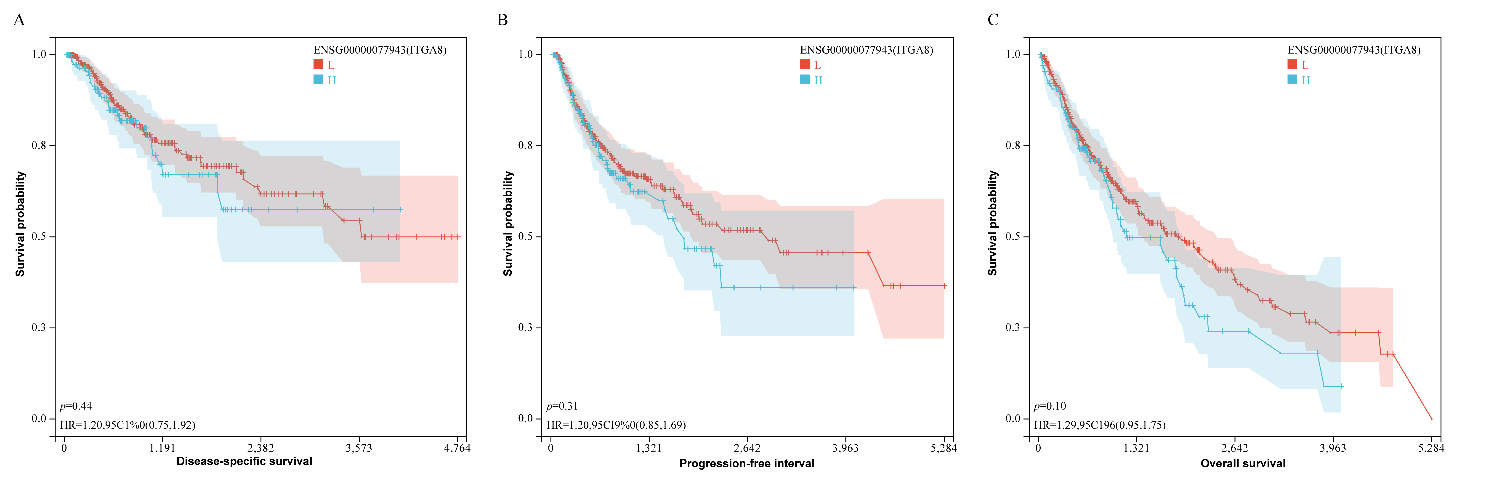


**Supplementary Figure 1** Patients with LUSC were divided into the high or low expression group according to ITGA8 expression and Kaplan–Meier survival analysis was performed for **(A)** disease-specific survival (*p* = 0.44), **(B)** progression-free interval (*p* = 0.31) and **(C)** overall survival (*p* = 0.10).


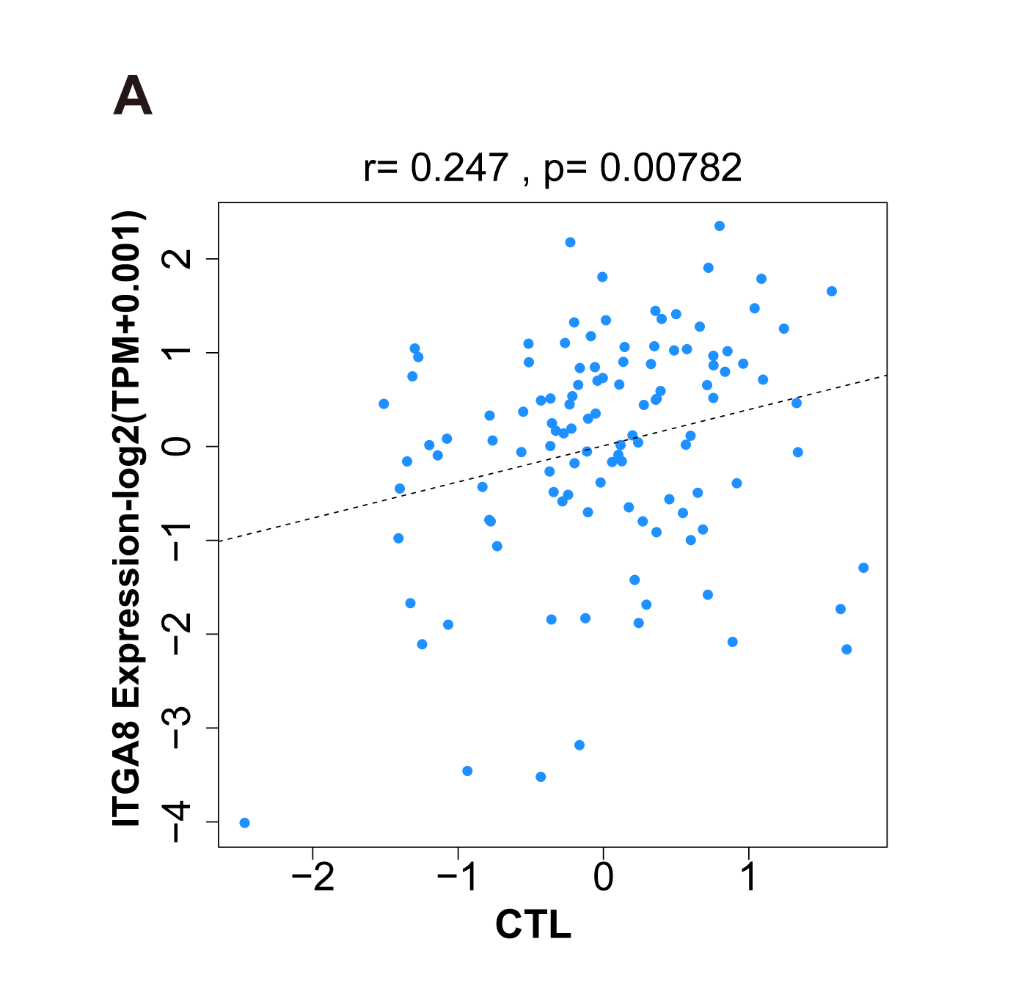


**Supplementary Figure 2 Correlation analyses between ITGA8 and cytotoxic T lymphocyte.** ITGA8-associated immune checkpoint blockade therapeutic effect based on ITGA8 expression level from the TCGA database using TIDE algorithm. The results showed that the score of CTL was positively correlated with ITGA8 expression; the higher the expression of ITGA8, the better the efficacy of immunotherapy. The expression level of ITGA8 gene was marked as Log 2(TPM+0.001) Scale.


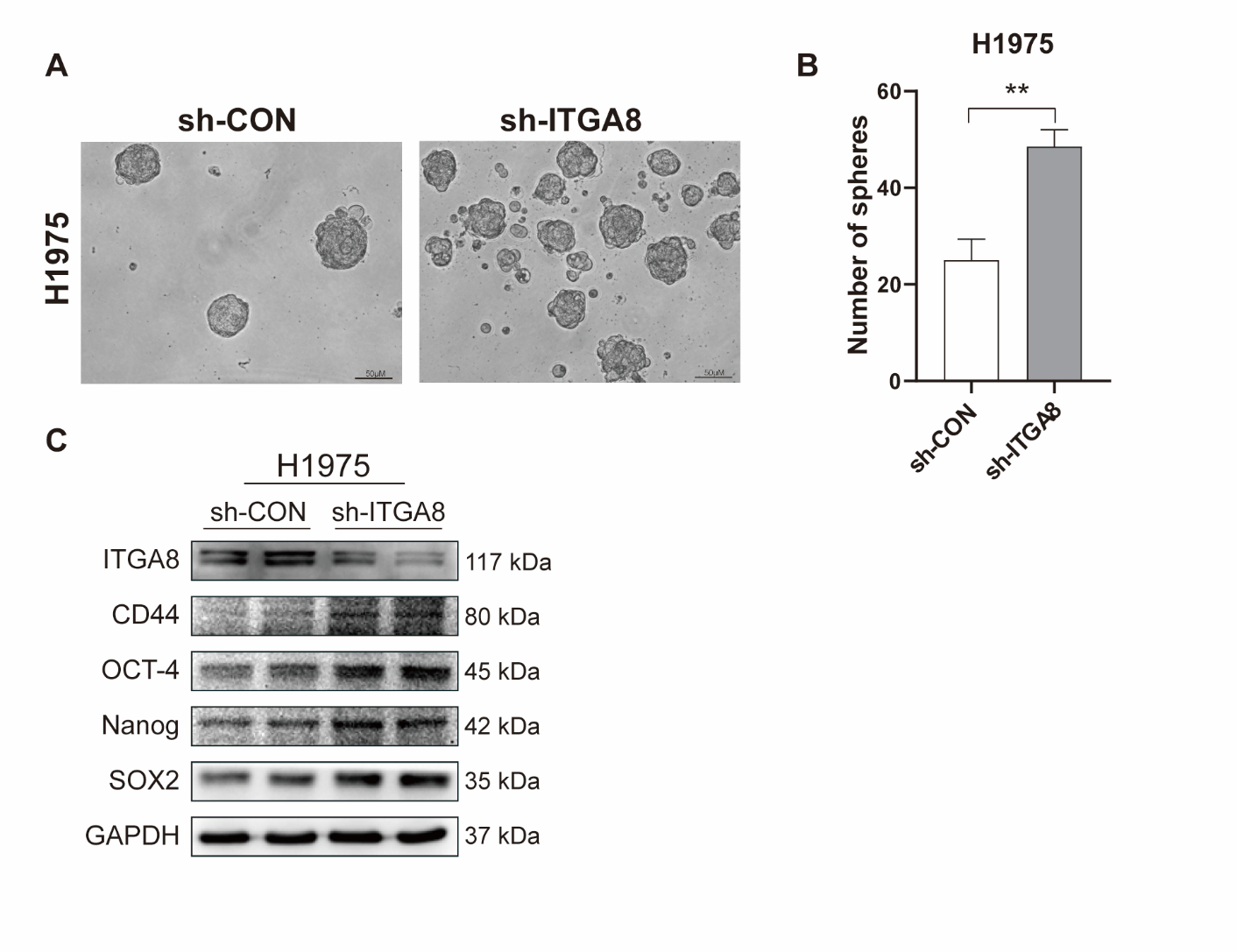


**Supplementary Figure 3 Experimental verification of the correlation between ITGA8 and cancer cell stemness. (A, B)** The sphere formation assay between sh-NC and sh-ITGA8 groups. **(C)** The expression of stemness markers between sh-NC and sh-ITGA8 in protein level. ***p* < 0.01.

**
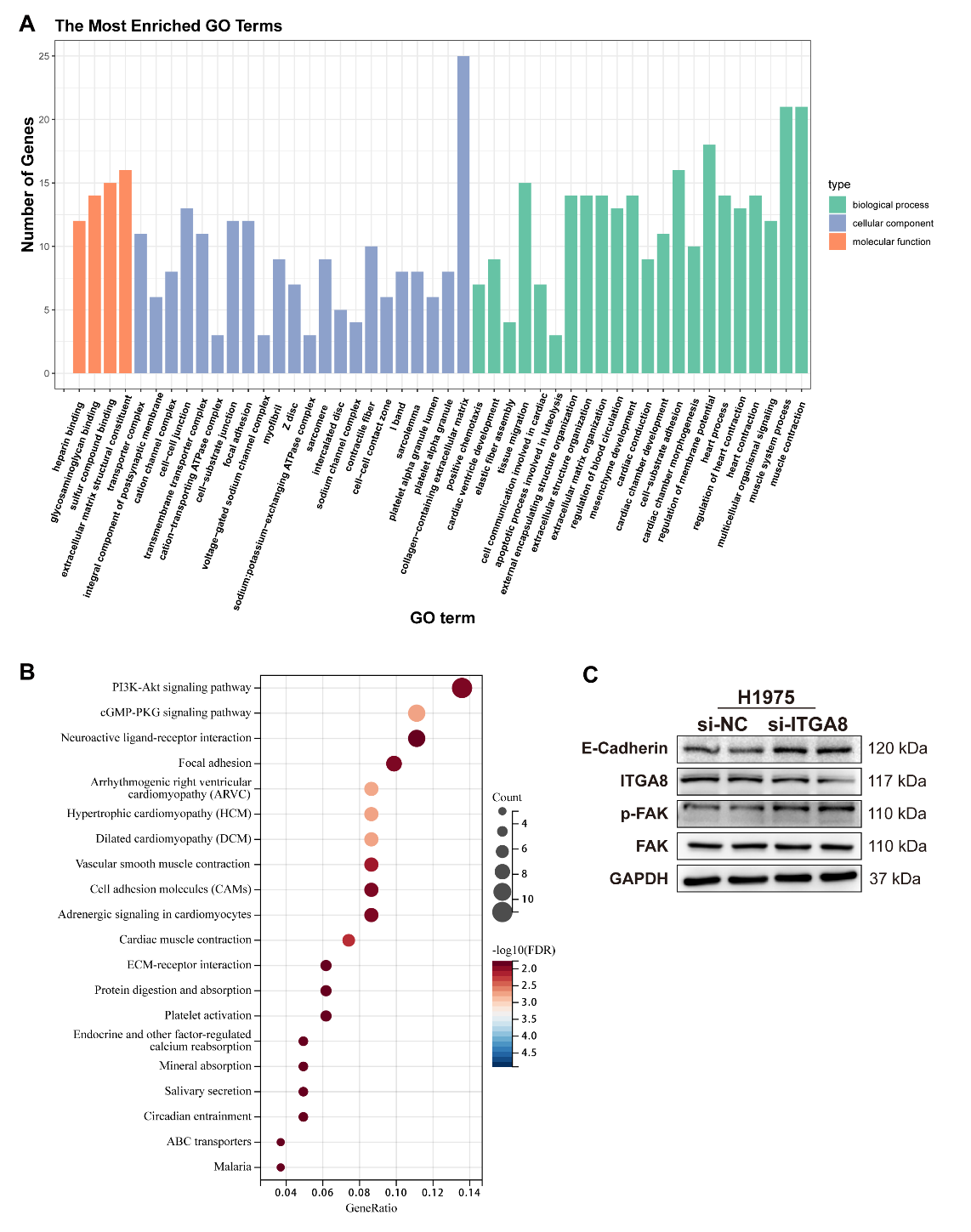
**

**Supplementary Figure 4 GO and KEGG enrichment analysis for genes, was shown to be highly correlated with ITGA8 through Person’s correlation analysis.** **(A)** The most enriched GO terms. **(B)** KEGG pathway enrichment. **(C)** The protein levels of E-Cadherin and FAK when ITGA8 was knockdown.


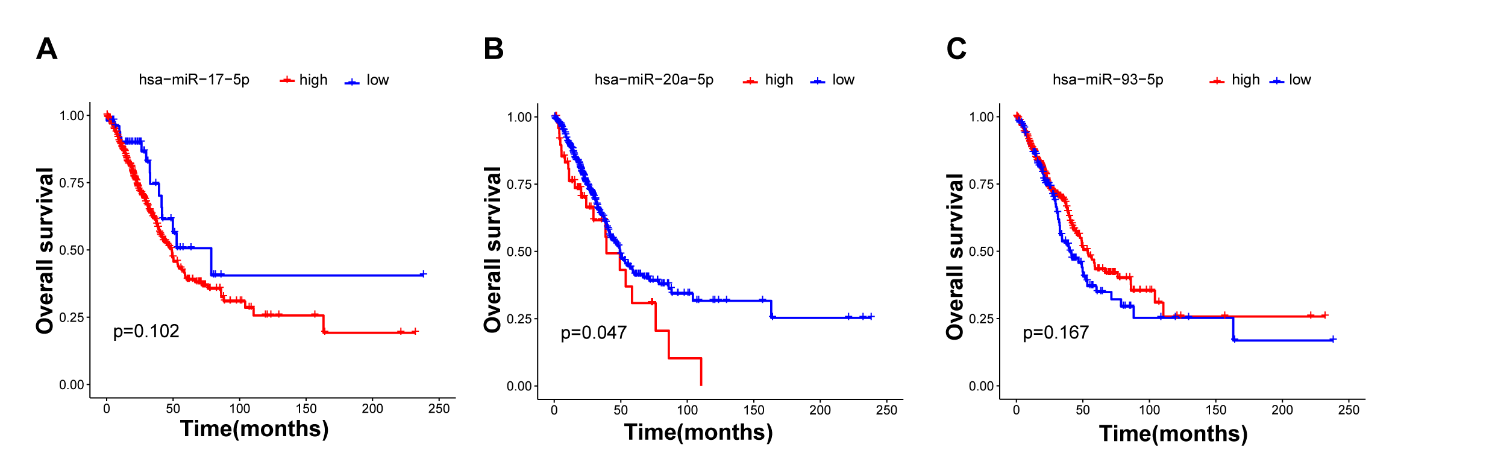


**Supplementary Figure 5** OS-based Kaplan–Meier survival analyses of **(A)** miR-17-5p (*p* = 0.102), **(B)** miR-20a-5p (*p* = 0.047), and **(C)** miR-93-5p (*p* = 0.167).


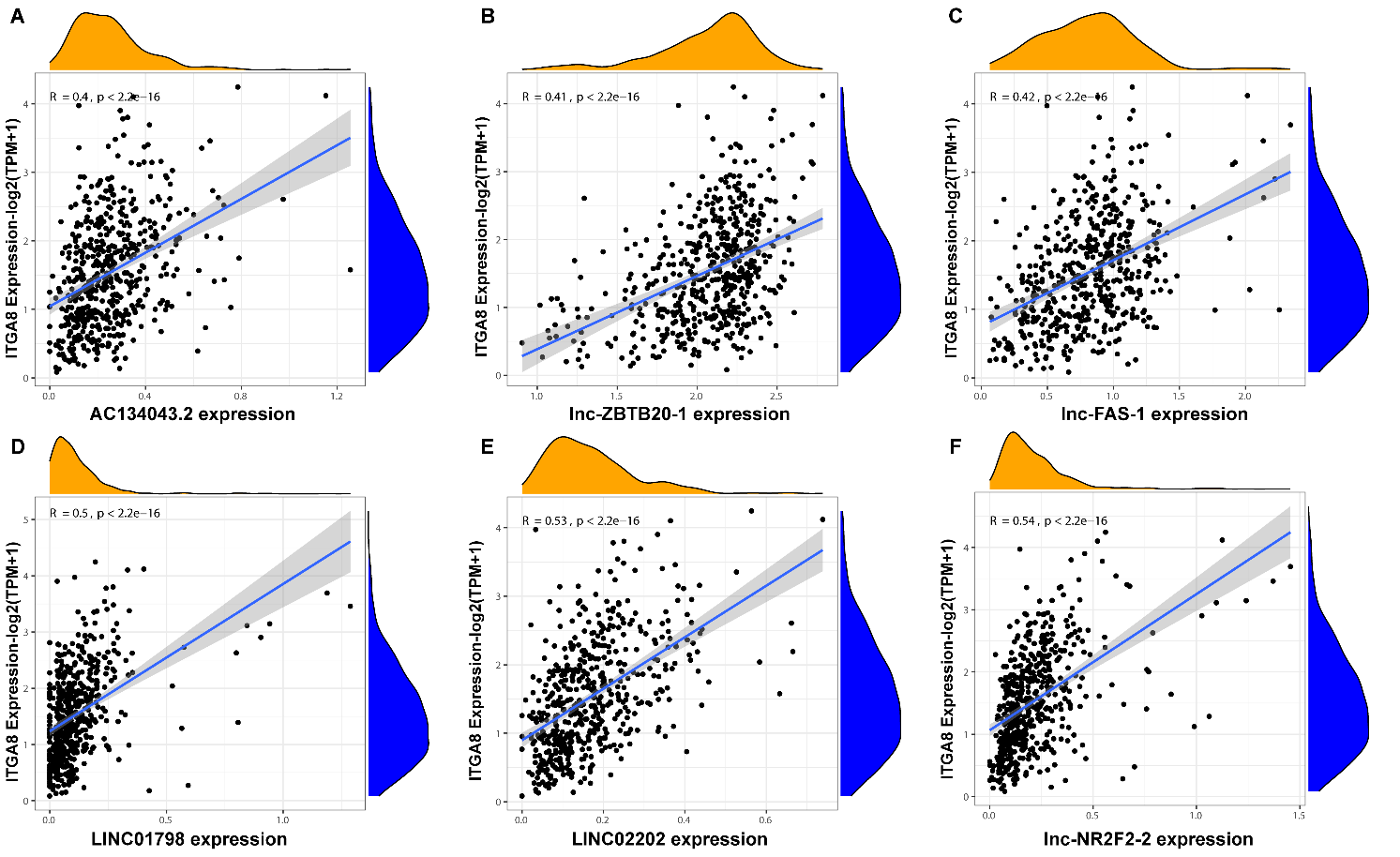


**Supplementary Figure 6 Correlation analyses between the expression of six lncRNAs and ITGA8. (A)** AC134043.2 (*r* = 0.4, *p* < 0.001), **(B)** lnc-ZBTB20-1 (*r* = 0.41, *p* < 0.001), **(C)** lnc-FAS-1 (*r* = 0.42, *p* < 0.001), **(D)** LINC01798 (*r* = 0.5, *p* < 0.001), **(E)** LINC02202 (*r* = 0.53, *p* < 0.001), and **(F)** lnc-NR2F2-2 (*r* = 0.54, *p* < 0.001). The expression level of ITGA8 gene was marked as Log 2(TPM+1) Scale.


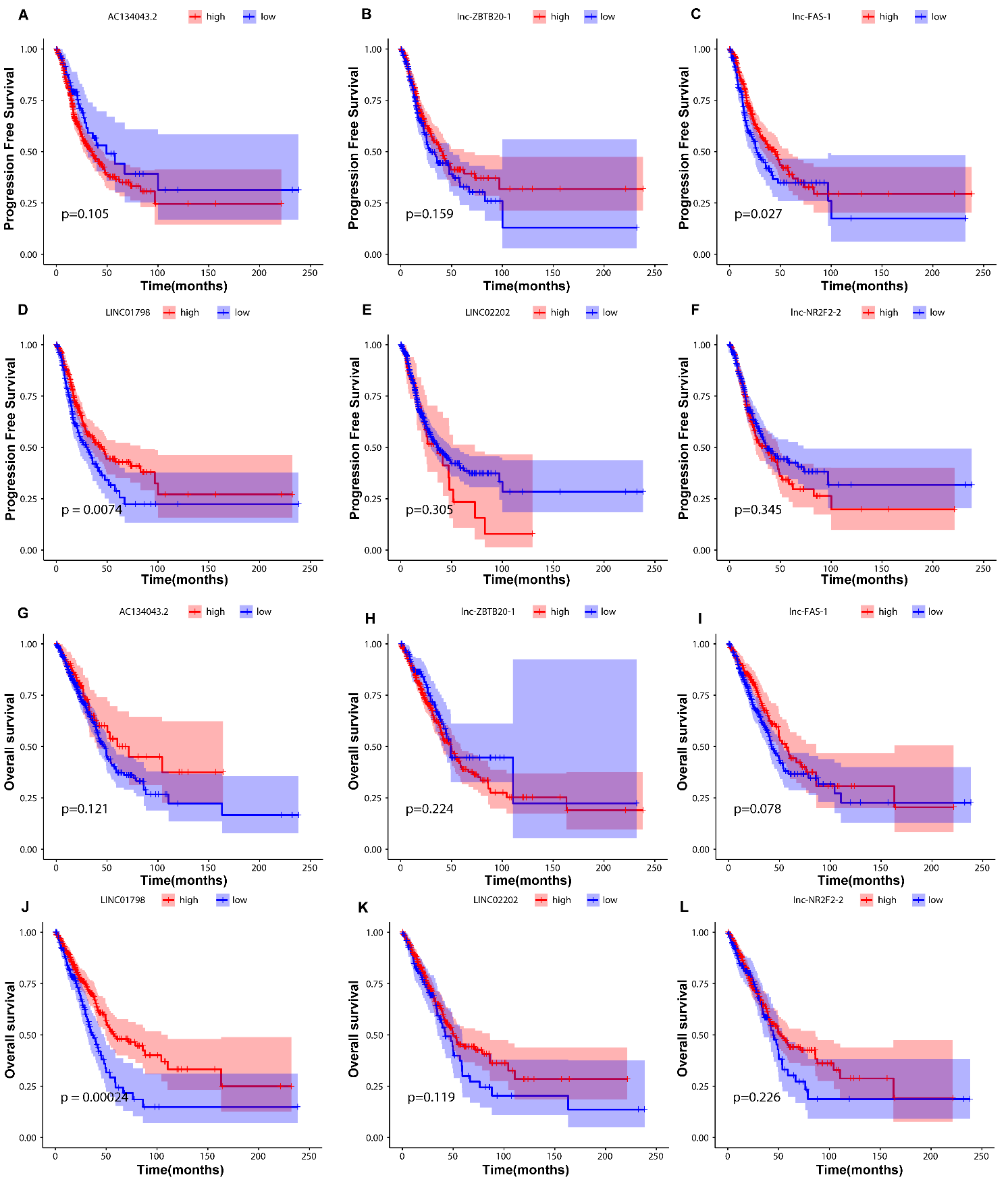


**Supplementary Figure 7 PFS-based and OS-based Kaplan–Meier survival analyses of the six lncRNAs**. PFS-based Kaplan–Meier survival analyses, **(A)** AC134043.2 (*p* = 0.105), **(B)** lnc-ZBTB20-1 (*p* = 0.159), **(C)** lnc-FAS-1 (*p* = 0.027), **(D)** LINC01798 (*p* = 0.0074), **(E)** LINC02202 (*p* = 0.305), and **(F)** lnc-NR2F2-2 (*p* = 0.345). OS-based Kaplan–Meier survival analyses, **(G)** AC134043.2 (*p* = 0.121), **(H)** lnc-ZBTB20-1 (*p* = 0.224), **(I)** lnc-FAS-1 (*p* = 0.078), **(J)** LINC01798 (*p* = 0.00024), **(K)** LINC02202 (*p* = 0.119), and **(L)** lnc-NR2F2-2 (*p* = 0.226).

## Supplementary Tables

| miRNA | ITGA8_cor | pvalue |
| --- | --- | --- |
| hsa-miR-17-5p | -0.3028695 | 4.36E-12 |
| hsa-miR-93-5p | -0.2835785 | 9.95E-11 |
| hsa-miR-20a-5p | -0.202069 | 4.84E-06 |

**Supplementary Table 1** Correlation analysis between the expression of miRNA and ITGA8

| miRNA name | LncRNA geneID | LncRNA Name |
| --- | --- | --- |
| hsa-miR-17-5p | ENSG00000240731 | AL139287.1 |
| hsa-miR-17-5p | ENSG00000215014 | AL645728.1 |
| hsa-miR-17-5p | ENSG00000117242 | PINK1-AS |
| hsa-miR-17-5p | ENSG00000240553 | AL031428.1 |
| hsa-miR-17-5p | ENSG00000281207 | SLFNL1-AS1 |
| hsa-miR-17-5p | ENSG00000226754 | AL606760.1 |
| hsa-miR-17-5p | ENSG00000275678 | AL133320.1 |
| hsa-miR-17-5p | ENSG00000223745 | CCDC18-AS1 |
| hsa-miR-17-5p | ENSG00000233184 | AC093157.1 |
| hsa-miR-17-5p | ENSG00000231734 | RP6-206I17.2 |
| hsa-miR-17-5p | ENSG00000228013 | IL6R-AS1 |
| hsa-miR-17-5p | ENSG00000260766 | RP11-226L15.5 |
| hsa-miR-17-5p | ENSG00000233693 | AL357568.1 |
| hsa-miR-17-5p | ENSG00000272205 | AL451050.2 |
| hsa-miR-17-5p | ENSG00000273160 | AL359962.2 |
| hsa-miR-17-5p | ENSG00000229989 | MIR181A1HG |
| hsa-miR-17-5p | ENSG00000281406 | BLACAT1 |
| hsa-miR-17-5p | ENSG00000237481 | AL117350.1 |
| hsa-miR-17-5p | ENSG00000273035 | AC007684.1 |
| hsa-miR-17-5p | ENSG00000212978 | AC016747.1 |
| hsa-miR-17-5p | ENSG00000232046 | LINC01798 |
| hsa-miR-17-5p | ENSG00000239322 | ATP6V1B1-AS1 |
| hsa-miR-17-5p | ENSG00000224189 | HAGLR |
| hsa-miR-17-5p | ENSG00000227308 | AC009502.1 |
| hsa-miR-17-5p | ENSG00000279809 | AC005538.2 |
| hsa-miR-17-5p | ENSG00000227252 | AC105760.2 |
| hsa-miR-17-5p | ENSG00000234949 | AC104667.2 |
| hsa-miR-17-5p | ENSG00000227110 | LMCD1-AS1 |
| hsa-miR-17-5p | ENSG00000225733 | FGD5-AS1 |
| hsa-miR-17-5p | ENSG00000280173 | AC104447.1 |
| hsa-miR-17-5p | ENSG00000243410 | PSMD6-AS1 |
| hsa-miR-17-5p | ENSG00000272610 | MAGI1-IT1 |
| hsa-miR-17-5p | ENSG00000259976 | AC093010.3 |
| hsa-miR-17-5p | ENSG00000273033 | LINC02035 |
| hsa-miR-17-5p | ENSG00000240086 | AC128689.1 |
| hsa-miR-17-5p | ENSG00000279891 | AC022498.2 |
| hsa-miR-17-5p | ENSG00000196810 | CTBP1-AS2 |
| hsa-miR-17-5p | ENSG00000273247 | AC097376.2 |
| hsa-miR-17-5p | ENSG00000273472 | AC096733.2 |
| hsa-miR-17-5p | ENSG00000250131 | AC078881.1 |
| hsa-miR-17-5p | ENSG00000177822 | AC098864.1 |
| hsa-miR-17-5p | ENSG00000248131 | LINC01194 |
| hsa-miR-17-5p | ENSG00000248092 | NNT-AS1 |
| hsa-miR-17-5p | ENSG00000152931 | PART1 |
| hsa-miR-17-5p | ENSG00000280187 | AC022107.1 |
| hsa-miR-17-5p | ENSG00000249042 | AC008771.1 |
| hsa-miR-17-5p | ENSG00000247572 | CKMT2-AS1 |
| hsa-miR-17-5p | ENSG00000250874 | AC010595.1 |
| hsa-miR-17-5p | ENSG00000247402 | AC099487.1 |
| hsa-miR-17-5p | ENSG00000224032 | EPB41L4A-AS1 |
| hsa-miR-17-5p | ENSG00000230551 | AC021078.1 |
| hsa-miR-17-5p | ENSG00000245812 | LINC02202 |
| hsa-miR-17-5p | ENSG00000279204 | AC134043.2 |
| hsa-miR-17-5p | ENSG00000261211 | AL031123.2 |
| hsa-miR-17-5p | ENSG00000231074 | HCG18 |
| hsa-miR-17-5p | ENSG00000206337 | HCP5 |
| hsa-miR-17-5p | ENSG00000261068 | AL512274.1 |
| hsa-miR-17-5p | ENSG00000231113 | AL035587.1 |
| hsa-miR-17-5p | ENSG00000273151 | AC073957.3 |
| hsa-miR-17-5p | ENSG00000234432 | AC092171.3 |
| hsa-miR-17-5p | ENSG00000273084 | AC092171.5 |
| hsa-miR-17-5p | ENSG00000279048 | AC080080.1 |
| hsa-miR-17-5p | ENSG00000233834 | AC005083.1 |
| hsa-miR-17-5p | ENSG00000238033 | AC002480.4 |
| hsa-miR-17-5p | ENSG00000233429 | HOTAIRM1 |
| hsa-miR-17-5p | ENSG00000253552 | HOXA-AS2 |
| hsa-miR-17-5p | ENSG00000196295 | AC005154.1 |
| hsa-miR-17-5p | ENSG00000232817 | AC073188.4 |
| hsa-miR-17-5p | ENSG00000272686 | AC006333.2 |
| hsa-miR-17-5p | ENSG00000273270 | AC090114.2 |
| hsa-miR-17-5p | ENSG00000270823 | AC007938.2 |
| hsa-miR-17-5p | ENSG00000261455 | LINC01003 |
| hsa-miR-17-5p | ENSG00000231419 | LINC00689 |
| hsa-miR-17-5p | ENSG00000253174 | AC009630.2 |
| hsa-miR-17-5p | ENSG00000228801 | AC064807.1 |
| hsa-miR-17-5p | ENSG00000253190 | AC084082.1 |
| hsa-miR-17-5p | ENSG00000253395 | AP003469.2 |
| hsa-miR-17-5p | ENSG00000253669 | GASAL1 |
| hsa-miR-17-5p | ENSG00000249859 | PVT1 |
| hsa-miR-17-5p | ENSG00000280303 | ERICD |
| hsa-miR-17-5p | ENSG00000232104 | RFX3-AS1 |
| hsa-miR-17-5p | ENSG00000260912 | AL158206.1 |
| hsa-miR-17-5p | ENSG00000281649 | EBLN3P |
| hsa-miR-17-5p | ENSG00000261094 | AC007066.2 |
| hsa-miR-17-5p | ENSG00000203993 | ARRDC1-AS1 |
| hsa-miR-17-5p | ENSG00000239665 | AL157392.3 |
| hsa-miR-17-5p | ENSG00000185904 | LINC00839 |
| hsa-miR-17-5p | ENSG00000226200 | SGMS1-AS1 |
| hsa-miR-17-5p | ENSG00000223502 | AL731537.1 |
| hsa-miR-17-5p | ENSG00000235931 | LINC01553 |
| hsa-miR-17-5p | ENSG00000233871 | DLG5-AS1 |
| hsa-miR-17-5p | ENSG00000230091 | TMEM254-AS1 |
| hsa-miR-17-5p | ENSG00000223482 | NUTM2A-AS1 |
| hsa-miR-17-5p | ENSG00000261438 | AL157394.1 |
| hsa-miR-17-5p | ENSG00000235823 | OLMALINC |
| hsa-miR-17-5p | ENSG00000269609 | RPARP-AS1 |
| hsa-miR-17-5p | ENSG00000130600 | H19 |
| hsa-miR-17-5p | ENSG00000269821 | KCNQ1OT1 |
| hsa-miR-17-5p | ENSG00000285338 | AC091564.7 |
| hsa-miR-17-5p | ENSG00000281880 | PAUPAR |
| hsa-miR-17-5p | ENSG00000245532 | NEAT1 |
| hsa-miR-17-5p | ENSG00000251562 | MALAT1 |
| hsa-miR-17-5p | ENSG00000258297 | AP001157.1 |
| hsa-miR-17-5p | ENSG00000255507 | AP003031.2 |
| hsa-miR-17-5p | ENSG00000255409 | RSF1-IT1 |
| hsa-miR-17-5p | ENSG00000279900 | AP001767.4 |
| hsa-miR-17-5p | ENSG00000272917 | AC010186.4 |
| hsa-miR-17-5p | ENSG00000284634 | AC092821.3 |
| hsa-miR-17-5p | ENSG00000247498 | GPRC5D-AS1 |
| hsa-miR-17-5p | ENSG00000275097 | AC024940.6 |
| hsa-miR-17-5p | ENSG00000276900 | AC023157.3 |
| hsa-miR-17-5p | ENSG00000257337 | AC068888.1 |
| hsa-miR-17-5p | ENSG00000228630 | HOTAIR |
| hsa-miR-17-5p | ENSG00000276853 | AC026124.2 |
| hsa-miR-17-5p | ENSG00000280088 | AC126474.2 |
| hsa-miR-17-5p | ENSG00000281344 | HELLPAR |
| hsa-miR-17-5p | ENSG00000280120 | AC073857.1 |
| hsa-miR-17-5p | ENSG00000256092 | AC137767.1 |
| hsa-miR-17-5p | ENSG00000278112 | AC145423.3 |
| hsa-miR-17-5p | ENSG00000279466 | AC073911.2 |
| hsa-miR-17-5p | ENSG00000278291 | AL161772.1 |
| hsa-miR-17-5p | ENSG00000176124 | DLEU1 |
| hsa-miR-17-5p | ENSG00000261553 | AL137782.1 |
| hsa-miR-17-5p | ENSG00000259321 | AL136295.2 |
| hsa-miR-17-5p | ENSG00000257621 | PSMA3-AS1 |
| hsa-miR-17-5p | ENSG00000258301 | VASH1-AS1 |
| hsa-miR-17-5p | ENSG00000258593 | AL583810.1 |
| hsa-miR-17-5p | ENSG00000257151 | PWAR6 |
| hsa-miR-17-5p | ENSG00000224078 | SNHG14 |
| hsa-miR-17-5p | ENSG00000259488 | AC023355.1 |
| hsa-miR-17-5p | ENSG00000244879 | GABPB1-AS1 |
| hsa-miR-17-5p | ENSG00000259476 | AC018904.2 |
| hsa-miR-17-5p | ENSG00000259771 | AC092756.1 |
| hsa-miR-17-5p | ENSG00000259248 | USP3-AS1 |
| hsa-miR-17-5p | ENSG00000281183 | NPTN-IT1 |
| hsa-miR-17-5p | ENSG00000260274 | AC068338.2 |
| hsa-miR-17-5p | ENSG00000278600 | AC015871.3 |
| hsa-miR-17-5p | ENSG00000259642 | ST20-AS1 |
| hsa-miR-17-5p | ENSG00000259416 | AC021739.3 |
| hsa-miR-17-5p | ENSG00000259275 | AC087477.2 |
| hsa-miR-17-5p | ENSG00000259583 | AC015712.2 |
| hsa-miR-17-5p | ENSG00000282907 | Z98883.1 |
| hsa-miR-17-5p | ENSG00000263033 | AC007220.1 |
| hsa-miR-17-5p | ENSG00000260280 | SLX1B-SULT1A4 |
| hsa-miR-17-5p | ENSG00000279789 | AC120114.4 |
| hsa-miR-17-5p | ENSG00000213599 | SLX1A-SULT1A3 |
| hsa-miR-17-5p | ENSG00000239791 | AC002310.2 |
| hsa-miR-17-5p | ENSG00000278133 | AC135050.6 |
| hsa-miR-17-5p | ENSG00000278928 | AC136621.1 |
| hsa-miR-17-5p | ENSG00000261519 | AC010542.4 |
| hsa-miR-17-5p | ENSG00000270165 | AC010530.1 |
| hsa-miR-17-5p | ENSG00000259768 | AC004943.2 |
| hsa-miR-17-5p | ENSG00000271009 | AC116667.1 |
| hsa-miR-17-5p | ENSG00000260816 | AC027279.1 |
| hsa-miR-17-5p | ENSG00000261175 | LINC02188 |
| hsa-miR-17-5p | ENSG00000279432 | AC015799.1 |
| hsa-miR-17-5p | ENSG00000262251 | AC087388.1 |
| hsa-miR-17-5p | ENSG00000277511 | AC116407.2 |
| hsa-miR-17-5p | ENSG00000265139 | AC005899.3 |
| hsa-miR-17-5p | ENSG00000278954 | AC130686.1 |
| hsa-miR-17-5p | ENSG00000244649 | LINC02086 |
| hsa-miR-17-5p | ENSG00000279089 | AC005839.1 |
| hsa-miR-17-5p | ENSG00000279281 | AC015883.1 |
| hsa-miR-17-5p | ENSG00000224738 | AC099850.1 |
| hsa-miR-17-5p | ENSG00000267416 | AC025048.4 |
| hsa-miR-17-5p | ENSG00000215769 | ARHGAP27P1-BPTFP1-KPNA2P3 |
| hsa-miR-17-5p | ENSG00000279573 | AC134407.2 |
| hsa-miR-17-5p | ENSG00000163597 | SNHG16 |
| hsa-miR-17-5p | ENSG00000234912 | SNHG20 |
| hsa-miR-17-5p | ENSG00000279187 | AC027601.5 |
| hsa-miR-17-5p | ENSG00000279066 | HEXDC-IT1 |
| hsa-miR-17-5p | ENSG00000262652 | AC124283.3 |
| hsa-miR-17-5p | ENSG00000263884 | AP000845.1 |
| hsa-miR-17-5p | ENSG00000266805 | AP005432.1 |
| hsa-miR-17-5p | ENSG00000268573 | AC011815.1 |
| hsa-miR-17-5p | ENSG00000260578 | AC110597.1 |
| hsa-miR-17-5p | ENSG00000267287 | AC068473.3 |
| hsa-miR-17-5p | ENSG00000279203 | AC005785.2 |
| hsa-miR-17-5p | ENSG00000279425 | AC092279.2 |
| hsa-miR-17-5p | ENSG00000280106 | AC008555.8 |
| hsa-miR-17-5p | ENSG00000267254 | AC020928.1 |
| hsa-miR-17-5p | ENSG00000267152 | AC093227.1 |
| hsa-miR-17-5p | ENSG00000279539 | AC006486.2 |
| hsa-miR-17-5p | ENSG00000186019 | AC021092.1 |
| hsa-miR-17-5p | ENSG00000279095 | AC243964.3 |
| hsa-miR-17-5p | ENSG00000260160 | AC011468.1 |
| hsa-miR-17-5p | ENSG00000269825 | AC022150.4 |
| hsa-miR-17-5p | ENSG00000267265 | AC011476.3 |
| hsa-miR-17-5p | ENSG00000276570 | AC010327.5 |
| hsa-miR-17-5p | ENSG00000268205 | AC005261.1 |
| hsa-miR-17-5p | ENSG00000176593 | AC008969.1 |
| hsa-miR-17-5p | ENSG00000228293 | AL049712.1 |
| hsa-miR-17-5p | ENSG00000277425 | AL121890.4 |
| hsa-miR-17-5p | ENSG00000277938 | AL035252.3 |
| hsa-miR-17-5p | ENSG00000277692 | AL121583.1 |
| hsa-miR-17-5p | ENSG00000260032 | NORAD |
| hsa-miR-17-5p | ENSG00000223891 | OSER1-AS1 |
| hsa-miR-17-5p | ENSG00000227477 | STK4-AS1 |
| hsa-miR-17-5p | ENSG00000280594 | BTG3-AS1 |
| hsa-miR-17-5p | ENSG00000261610 | AP000265.1 |
| hsa-miR-17-5p | ENSG00000280604 | AJ239328.1 |
| hsa-miR-17-5p | ENSG00000093100 | AC016026.1 |
| hsa-miR-17-5p | ENSG00000230513 | THAP7-AS1 |
| hsa-miR-17-5p | ENSG00000278657 | AC245452.5 |
| hsa-miR-17-5p | ENSG00000279217 | Z95114.1 |
| hsa-miR-17-5p | ENSG00000279738 | AL022311.1 |
| hsa-miR-17-5p | ENSG00000280434 | AL031595.3 |
| hsa-miR-17-5p | ENSG00000280011 | AL031595.2 |
| hsa-miR-17-5p | ENSG00000197182 | MIRLET7BHG |
| hsa-miR-17-5p | ENSG00000260822 | AC004656.1 |
| hsa-miR-17-5p | ENSG00000241886 | AC112496.1 |
| hsa-miR-17-5p | ENSG00000226310 | AL022157.1 |
| hsa-miR-17-5p | ENSG00000229807 | XIST |
| hsa-miR-17-5p | ENSG00000280195 | AC245140.2 |
| hsa-miR-17-5p | ENSG00000233864 | TTTY15 |
| hsa-miR-20a-5p | ENSG00000240731 | AL139287.1 |
| hsa-miR-20a-5p | ENSG00000215014 | AL645728.1 |
| hsa-miR-20a-5p | ENSG00000117242 | PINK1-AS |
| hsa-miR-20a-5p | ENSG00000240553 | AL031428.1 |
| hsa-miR-20a-5p | ENSG00000281207 | SLFNL1-AS1 |
| hsa-miR-20a-5p | ENSG00000226754 | AL606760.1 |
| hsa-miR-20a-5p | ENSG00000275678 | AL133320.1 |
| hsa-miR-20a-5p | ENSG00000223745 | CCDC18-AS1 |
| hsa-miR-20a-5p | ENSG00000233184 | AC093157.1 |
| hsa-miR-20a-5p | ENSG00000231734 | RP6-206I17.2 |
| hsa-miR-20a-5p | ENSG00000228013 | IL6R-AS1 |
| hsa-miR-20a-5p | ENSG00000260766 | RP11-226L15.5 |
| hsa-miR-20a-5p | ENSG00000233693 | AL357568.1 |
| hsa-miR-20a-5p | ENSG00000272205 | AL451050.2 |
| hsa-miR-20a-5p | ENSG00000273160 | AL359962.2 |
| hsa-miR-20a-5p | ENSG00000229989 | MIR181A1HG |
| hsa-miR-20a-5p | ENSG00000281406 | BLACAT1 |
| hsa-miR-20a-5p | ENSG00000237481 | AL117350.1 |
| hsa-miR-20a-5p | ENSG00000273035 | AC007684.1 |
| hsa-miR-20a-5p | ENSG00000212978 | AC016747.1 |
| hsa-miR-20a-5p | ENSG00000232046 | LINC01798 |
| hsa-miR-20a-5p | ENSG00000239322 | ATP6V1B1-AS1 |
| hsa-miR-20a-5p | ENSG00000224189 | HAGLR |
| hsa-miR-20a-5p | ENSG00000227308 | AC009502.1 |
| hsa-miR-20a-5p | ENSG00000279809 | AC005538.2 |
| hsa-miR-20a-5p | ENSG00000227252 | AC105760.2 |
| hsa-miR-20a-5p | ENSG00000234949 | AC104667.2 |
| hsa-miR-20a-5p | ENSG00000227110 | LMCD1-AS1 |
| hsa-miR-20a-5p | ENSG00000225733 | FGD5-AS1 |
| hsa-miR-20a-5p | ENSG00000280173 | AC104447.1 |
| hsa-miR-20a-5p | ENSG00000243410 | PSMD6-AS1 |
| hsa-miR-20a-5p | ENSG00000272610 | MAGI1-IT1 |
| hsa-miR-20a-5p | ENSG00000259976 | AC093010.3 |
| hsa-miR-20a-5p | ENSG00000273033 | LINC02035 |
| hsa-miR-20a-5p | ENSG00000240086 | AC128689.1 |
| hsa-miR-20a-5p | ENSG00000279891 | AC022498.2 |
| hsa-miR-20a-5p | ENSG00000196810 | CTBP1-AS2 |
| hsa-miR-20a-5p | ENSG00000273247 | AC097376.2 |
| hsa-miR-20a-5p | ENSG00000273472 | AC096733.2 |
| hsa-miR-20a-5p | ENSG00000250131 | AC078881.1 |
| hsa-miR-20a-5p | ENSG00000177822 | AC098864.1 |
| hsa-miR-20a-5p | ENSG00000248131 | LINC01194 |
| hsa-miR-20a-5p | ENSG00000248092 | NNT-AS1 |
| hsa-miR-20a-5p | ENSG00000152931 | PART1 |
| hsa-miR-20a-5p | ENSG00000280187 | AC022107.1 |
| hsa-miR-20a-5p | ENSG00000249042 | AC008771.1 |
| hsa-miR-20a-5p | ENSG00000247572 | CKMT2-AS1 |
| hsa-miR-20a-5p | ENSG00000250874 | AC010595.1 |
| hsa-miR-20a-5p | ENSG00000247402 | AC099487.1 |
| hsa-miR-20a-5p | ENSG00000224032 | EPB41L4A-AS1 |
| hsa-miR-20a-5p | ENSG00000230551 | AC021078.1 |
| hsa-miR-20a-5p | ENSG00000245812 | LINC02202 |
| hsa-miR-20a-5p | ENSG00000279204 | AC134043.2 |
| hsa-miR-20a-5p | ENSG00000261211 | AL031123.2 |
| hsa-miR-20a-5p | ENSG00000231074 | HCG18 |
| hsa-miR-20a-5p | ENSG00000206337 | HCP5 |
| hsa-miR-20a-5p | ENSG00000261068 | AL512274.1 |
| hsa-miR-20a-5p | ENSG00000231113 | AL035587.1 |
| hsa-miR-20a-5p | ENSG00000273151 | AC073957.3 |
| hsa-miR-20a-5p | ENSG00000234432 | AC092171.3 |
| hsa-miR-20a-5p | ENSG00000273084 | AC092171.5 |
| hsa-miR-20a-5p | ENSG00000279048 | AC080080.1 |
| hsa-miR-20a-5p | ENSG00000233834 | AC005083.1 |
| hsa-miR-20a-5p | ENSG00000238033 | AC002480.4 |
| hsa-miR-20a-5p | ENSG00000233429 | HOTAIRM1 |
| hsa-miR-20a-5p | ENSG00000253552 | HOXA-AS2 |
| hsa-miR-20a-5p | ENSG00000196295 | AC005154.1 |
| hsa-miR-20a-5p | ENSG00000232817 | AC073188.4 |
| hsa-miR-20a-5p | ENSG00000272686 | AC006333.2 |
| hsa-miR-20a-5p | ENSG00000273270 | AC090114.2 |
| hsa-miR-20a-5p | ENSG00000270823 | AC007938.2 |
| hsa-miR-20a-5p | ENSG00000261455 | LINC01003 |
| hsa-miR-20a-5p | ENSG00000231419 | LINC00689 |
| hsa-miR-20a-5p | ENSG00000253174 | AC009630.2 |
| hsa-miR-20a-5p | ENSG00000228801 | AC064807.1 |
| hsa-miR-20a-5p | ENSG00000253190 | AC084082.1 |
| hsa-miR-20a-5p | ENSG00000253395 | AP003469.2 |
| hsa-miR-20a-5p | ENSG00000253669 | GASAL1 |
| hsa-miR-20a-5p | ENSG00000249859 | PVT1 |
| hsa-miR-20a-5p | ENSG00000280303 | ERICD |
| hsa-miR-20a-5p | ENSG00000232104 | RFX3-AS1 |
| hsa-miR-20a-5p | ENSG00000260912 | AL158206.1 |
| hsa-miR-20a-5p | ENSG00000281649 | EBLN3P |
| hsa-miR-20a-5p | ENSG00000261094 | AC007066.2 |
| hsa-miR-20a-5p | ENSG00000203993 | ARRDC1-AS1 |
| hsa-miR-20a-5p | ENSG00000239665 | AL157392.3 |
| hsa-miR-20a-5p | ENSG00000185904 | LINC00839 |
| hsa-miR-20a-5p | ENSG00000226200 | SGMS1-AS1 |
| hsa-miR-20a-5p | ENSG00000223502 | AL731537.1 |
| hsa-miR-20a-5p | ENSG00000235931 | LINC01553 |
| hsa-miR-20a-5p | ENSG00000233871 | DLG5-AS1 |
| hsa-miR-20a-5p | ENSG00000230091 | TMEM254-AS1 |
| hsa-miR-20a-5p | ENSG00000223482 | NUTM2A-AS1 |
| hsa-miR-20a-5p | ENSG00000261438 | AL157394.1 |
| hsa-miR-20a-5p | ENSG00000235823 | OLMALINC |
| hsa-miR-20a-5p | ENSG00000269609 | RPARP-AS1 |
| hsa-miR-20a-5p | ENSG00000130600 | H19 |
| hsa-miR-20a-5p | ENSG00000269821 | KCNQ1OT1 |
| hsa-miR-20a-5p | ENSG00000285338 | AC091564.7 |
| hsa-miR-20a-5p | ENSG00000281880 | PAUPAR |
| hsa-miR-20a-5p | ENSG00000245532 | NEAT1 |
| hsa-miR-20a-5p | ENSG00000251562 | MALAT1 |
| hsa-miR-20a-5p | ENSG00000258297 | AP001157.1 |
| hsa-miR-20a-5p | ENSG00000255507 | AP003031.2 |
| hsa-miR-20a-5p | ENSG00000255409 | RSF1-IT1 |
| hsa-miR-20a-5p | ENSG00000279900 | AP001767.4 |
| hsa-miR-20a-5p | ENSG00000272917 | AC010186.4 |
| hsa-miR-20a-5p | ENSG00000284634 | AC092821.3 |
| hsa-miR-20a-5p | ENSG00000247498 | GPRC5D-AS1 |
| hsa-miR-20a-5p | ENSG00000275097 | AC024940.6 |
| hsa-miR-20a-5p | ENSG00000276900 | AC023157.3 |
| hsa-miR-20a-5p | ENSG00000257337 | AC068888.1 |
| hsa-miR-20a-5p | ENSG00000228630 | HOTAIR |
| hsa-miR-20a-5p | ENSG00000276853 | AC026124.2 |
| hsa-miR-20a-5p | ENSG00000280088 | AC126474.2 |
| hsa-miR-20a-5p | ENSG00000281344 | HELLPAR |
| hsa-miR-20a-5p | ENSG00000280120 | AC073857.1 |
| hsa-miR-20a-5p | ENSG00000256092 | AC137767.1 |
| hsa-miR-20a-5p | ENSG00000278112 | AC145423.3 |
| hsa-miR-20a-5p | ENSG00000279466 | AC073911.2 |
| hsa-miR-20a-5p | ENSG00000278291 | AL161772.1 |
| hsa-miR-20a-5p | ENSG00000176124 | DLEU1 |
| hsa-miR-20a-5p | ENSG00000261553 | AL137782.1 |
| hsa-miR-20a-5p | ENSG00000259321 | AL136295.2 |
| hsa-miR-20a-5p | ENSG00000257621 | PSMA3-AS1 |
| hsa-miR-20a-5p | ENSG00000258301 | VASH1-AS1 |
| hsa-miR-20a-5p | ENSG00000258593 | AL583810.1 |
| hsa-miR-20a-5p | ENSG00000257151 | PWAR6 |
| hsa-miR-20a-5p | ENSG00000224078 | SNHG14 |
| hsa-miR-20a-5p | ENSG00000259488 | AC023355.1 |
| hsa-miR-20a-5p | ENSG00000244879 | GABPB1-AS1 |
| hsa-miR-20a-5p | ENSG00000259476 | AC018904.2 |
| hsa-miR-20a-5p | ENSG00000259771 | AC092756.1 |
| hsa-miR-20a-5p | ENSG00000259248 | USP3-AS1 |
| hsa-miR-20a-5p | ENSG00000281183 | NPTN-IT1 |
| hsa-miR-20a-5p | ENSG00000260274 | AC068338.2 |
| hsa-miR-20a-5p | ENSG00000278600 | AC015871.3 |
| hsa-miR-20a-5p | ENSG00000259642 | ST20-AS1 |
| hsa-miR-20a-5p | ENSG00000259416 | AC021739.3 |
| hsa-miR-20a-5p | ENSG00000259275 | AC087477.2 |
| hsa-miR-20a-5p | ENSG00000259583 | AC015712.2 |
| hsa-miR-20a-5p | ENSG00000282907 | Z98883.1 |
| hsa-miR-20a-5p | ENSG00000263033 | AC007220.1 |
| hsa-miR-20a-5p | ENSG00000260280 | SLX1B-SULT1A4 |
| hsa-miR-20a-5p | ENSG00000279789 | AC120114.4 |
| hsa-miR-20a-5p | ENSG00000213599 | SLX1A-SULT1A3 |
| hsa-miR-20a-5p | ENSG00000239791 | AC002310.2 |
| hsa-miR-20a-5p | ENSG00000278133 | AC135050.6 |
| hsa-miR-20a-5p | ENSG00000278928 | AC136621.1 |
| hsa-miR-20a-5p | ENSG00000261519 | AC010542.4 |
| hsa-miR-20a-5p | ENSG00000270165 | AC010530.1 |
| hsa-miR-20a-5p | ENSG00000259768 | AC004943.2 |
| hsa-miR-20a-5p | ENSG00000271009 | AC116667.1 |
| hsa-miR-20a-5p | ENSG00000260816 | AC027279.1 |
| hsa-miR-20a-5p | ENSG00000261175 | LINC02188 |
| hsa-miR-20a-5p | ENSG00000279432 | AC015799.1 |
| hsa-miR-20a-5p | ENSG00000262251 | AC087388.1 |
| hsa-miR-20a-5p | ENSG00000277511 | AC116407.2 |
| hsa-miR-20a-5p | ENSG00000265139 | AC005899.3 |
| hsa-miR-20a-5p | ENSG00000278954 | AC130686.1 |
| hsa-miR-20a-5p | ENSG00000244649 | LINC02086 |
| hsa-miR-20a-5p | ENSG00000279089 | AC005839.1 |
| hsa-miR-20a-5p | ENSG00000279281 | AC015883.1 |
| hsa-miR-20a-5p | ENSG00000224738 | AC099850.1 |
| hsa-miR-20a-5p | ENSG00000267416 | AC025048.4 |
| hsa-miR-20a-5p | ENSG00000215769 | ARHGAP27P1-BPTFP1-KPNA2P3 |
| hsa-miR-20a-5p | ENSG00000279573 | AC134407.2 |
| hsa-miR-20a-5p | ENSG00000163597 | SNHG16 |
| hsa-miR-20a-5p | ENSG00000234912 | SNHG20 |
| hsa-miR-20a-5p | ENSG00000279187 | AC027601.5 |
| hsa-miR-20a-5p | ENSG00000279066 | HEXDC-IT1 |
| hsa-miR-20a-5p | ENSG00000262652 | AC124283.3 |
| hsa-miR-20a-5p | ENSG00000263884 | AP000845.1 |
| hsa-miR-20a-5p | ENSG00000266805 | AP005432.1 |
| hsa-miR-20a-5p | ENSG00000268573 | AC011815.1 |
| hsa-miR-20a-5p | ENSG00000260578 | AC110597.1 |
| hsa-miR-20a-5p | ENSG00000267287 | AC068473.3 |
| hsa-miR-20a-5p | ENSG00000279203 | AC005785.2 |
| hsa-miR-20a-5p | ENSG00000279425 | AC092279.2 |
| hsa-miR-20a-5p | ENSG00000280106 | AC008555.8 |
| hsa-miR-20a-5p | ENSG00000267254 | AC020928.1 |
| hsa-miR-20a-5p | ENSG00000267152 | AC093227.1 |
| hsa-miR-20a-5p | ENSG00000279539 | AC006486.2 |
| hsa-miR-20a-5p | ENSG00000186019 | AC021092.1 |
| hsa-miR-20a-5p | ENSG00000279095 | AC243964.3 |
| hsa-miR-20a-5p | ENSG00000260160 | AC011468.1 |
| hsa-miR-20a-5p | ENSG00000269825 | AC022150.4 |
| hsa-miR-20a-5p | ENSG00000267265 | AC011476.3 |
| hsa-miR-20a-5p | ENSG00000276570 | AC010327.5 |
| hsa-miR-20a-5p | ENSG00000268205 | AC005261.1 |
| hsa-miR-20a-5p | ENSG00000176593 | AC008969.1 |
| hsa-miR-20a-5p | ENSG00000228293 | AL049712.1 |
| hsa-miR-20a-5p | ENSG00000277425 | AL121890.4 |
| hsa-miR-20a-5p | ENSG00000277938 | AL035252.3 |
| hsa-miR-20a-5p | ENSG00000277692 | AL121583.1 |
| hsa-miR-20a-5p | ENSG00000260032 | NORAD |
| hsa-miR-20a-5p | ENSG00000223891 | OSER1-AS1 |
| hsa-miR-20a-5p | ENSG00000227477 | STK4-AS1 |
| hsa-miR-20a-5p | ENSG00000280594 | BTG3-AS1 |
| hsa-miR-20a-5p | ENSG00000261610 | AP000265.1 |
| hsa-miR-20a-5p | ENSG00000280604 | AJ239328.1 |
| hsa-miR-20a-5p | ENSG00000093100 | AC016026.1 |
| hsa-miR-20a-5p | ENSG00000230513 | THAP7-AS1 |
| hsa-miR-20a-5p | ENSG00000278657 | AC245452.5 |
| hsa-miR-20a-5p | ENSG00000279217 | Z95114.1 |
| hsa-miR-20a-5p | ENSG00000279738 | AL022311.1 |
| hsa-miR-20a-5p | ENSG00000280434 | AL031595.3 |
| hsa-miR-20a-5p | ENSG00000280011 | AL031595.2 |
| hsa-miR-20a-5p | ENSG00000197182 | MIRLET7BHG |
| hsa-miR-20a-5p | ENSG00000260822 | AC004656.1 |
| hsa-miR-20a-5p | ENSG00000241886 | AC112496.1 |
| hsa-miR-20a-5p | ENSG00000226310 | AL022157.1 |
| hsa-miR-20a-5p | ENSG00000229807 | XIST |
| hsa-miR-20a-5p | ENSG00000280195 | AC245140.2 |
| hsa-miR-20a-5p | ENSG00000233864 | TTTY15 |
| hsa-miR-93-5p | ENSG00000240731 | AL139287.1 |
| hsa-miR-93-5p | ENSG00000215014 | AL645728.1 |
| hsa-miR-93-5p | ENSG00000117242 | PINK1-AS |
| hsa-miR-93-5p | ENSG00000240553 | AL031428.1 |
| hsa-miR-93-5p | ENSG00000281207 | SLFNL1-AS1 |
| hsa-miR-93-5p | ENSG00000226754 | AL606760.1 |
| hsa-miR-93-5p | ENSG00000275678 | AL133320.1 |
| hsa-miR-93-5p | ENSG00000223745 | CCDC18-AS1 |
| hsa-miR-93-5p | ENSG00000233184 | AC093157.1 |
| hsa-miR-93-5p | ENSG00000231734 | RP6-206I17.2 |
| hsa-miR-93-5p | ENSG00000228013 | IL6R-AS1 |
| hsa-miR-93-5p | ENSG00000260766 | RP11-226L15.5 |
| hsa-miR-93-5p | ENSG00000233693 | AL357568.1 |
| hsa-miR-93-5p | ENSG00000272205 | AL451050.2 |
| hsa-miR-93-5p | ENSG00000273160 | AL359962.2 |
| hsa-miR-93-5p | ENSG00000229989 | MIR181A1HG |
| hsa-miR-93-5p | ENSG00000281406 | BLACAT1 |
| hsa-miR-93-5p | ENSG00000237481 | AL117350.1 |
| hsa-miR-93-5p | ENSG00000273035 | AC007684.1 |
| hsa-miR-93-5p | ENSG00000212978 | AC016747.1 |
| hsa-miR-93-5p | ENSG00000232046 | LINC01798 |
| hsa-miR-93-5p | ENSG00000239322 | ATP6V1B1-AS1 |
| hsa-miR-93-5p | ENSG00000224189 | HAGLR |
| hsa-miR-93-5p | ENSG00000227308 | AC009502.1 |
| hsa-miR-93-5p | ENSG00000279809 | AC005538.2 |
| hsa-miR-93-5p | ENSG00000227252 | AC105760.2 |
| hsa-miR-93-5p | ENSG00000234949 | AC104667.2 |
| hsa-miR-93-5p | ENSG00000227110 | LMCD1-AS1 |
| hsa-miR-93-5p | ENSG00000225733 | FGD5-AS1 |
| hsa-miR-93-5p | ENSG00000280173 | AC104447.1 |
| hsa-miR-93-5p | ENSG00000243410 | PSMD6-AS1 |
| hsa-miR-93-5p | ENSG00000272610 | MAGI1-IT1 |
| hsa-miR-93-5p | ENSG00000259976 | AC093010.3 |
| hsa-miR-93-5p | ENSG00000273033 | LINC02035 |
| hsa-miR-93-5p | ENSG00000240086 | AC128689.1 |
| hsa-miR-93-5p | ENSG00000279891 | AC022498.2 |
| hsa-miR-93-5p | ENSG00000196810 | CTBP1-AS2 |
| hsa-miR-93-5p | ENSG00000273247 | AC097376.2 |
| hsa-miR-93-5p | ENSG00000273472 | AC096733.2 |
| hsa-miR-93-5p | ENSG00000250131 | AC078881.1 |
| hsa-miR-93-5p | ENSG00000177822 | AC098864.1 |
| hsa-miR-93-5p | ENSG00000248131 | LINC01194 |
| hsa-miR-93-5p | ENSG00000248092 | NNT-AS1 |
| hsa-miR-93-5p | ENSG00000152931 | PART1 |
| hsa-miR-93-5p | ENSG00000280187 | AC022107.1 |
| hsa-miR-93-5p | ENSG00000249042 | AC008771.1 |
| hsa-miR-93-5p | ENSG00000247572 | CKMT2-AS1 |
| hsa-miR-93-5p | ENSG00000250874 | AC010595.1 |
| hsa-miR-93-5p | ENSG00000247402 | AC099487.1 |
| hsa-miR-93-5p | ENSG00000224032 | EPB41L4A-AS1 |
| hsa-miR-93-5p | ENSG00000230551 | AC021078.1 |
| hsa-miR-93-5p | ENSG00000245812 | LINC02202 |
| hsa-miR-93-5p | ENSG00000279204 | AC134043.2 |
| hsa-miR-93-5p | ENSG00000261211 | AL031123.2 |
| hsa-miR-93-5p | ENSG00000231074 | HCG18 |
| hsa-miR-93-5p | ENSG00000206337 | HCP5 |
| hsa-miR-93-5p | ENSG00000261068 | AL512274.1 |
| hsa-miR-93-5p | ENSG00000231113 | AL035587.1 |
| hsa-miR-93-5p | ENSG00000273151 | AC073957.3 |
| hsa-miR-93-5p | ENSG00000234432 | AC092171.3 |
| hsa-miR-93-5p | ENSG00000273084 | AC092171.5 |
| hsa-miR-93-5p | ENSG00000279048 | AC080080.1 |
| hsa-miR-93-5p | ENSG00000233834 | AC005083.1 |
| hsa-miR-93-5p | ENSG00000238033 | AC002480.4 |
| hsa-miR-93-5p | ENSG00000233429 | HOTAIRM1 |
| hsa-miR-93-5p | ENSG00000253552 | HOXA-AS2 |
| hsa-miR-93-5p | ENSG00000196295 | AC005154.1 |
| hsa-miR-93-5p | ENSG00000232817 | AC073188.4 |
| hsa-miR-93-5p | ENSG00000272686 | AC006333.2 |
| hsa-miR-93-5p | ENSG00000273270 | AC090114.2 |
| hsa-miR-93-5p | ENSG00000270823 | AC007938.2 |
| hsa-miR-93-5p | ENSG00000261455 | LINC01003 |
| hsa-miR-93-5p | ENSG00000231419 | LINC00689 |
| hsa-miR-93-5p | ENSG00000253174 | AC009630.2 |
| hsa-miR-93-5p | ENSG00000228801 | AC064807.1 |
| hsa-miR-93-5p | ENSG00000253190 | AC084082.1 |
| hsa-miR-93-5p | ENSG00000253395 | AP003469.2 |
| hsa-miR-93-5p | ENSG00000253669 | GASAL1 |
| hsa-miR-93-5p | ENSG00000249859 | PVT1 |
| hsa-miR-93-5p | ENSG00000280303 | ERICD |
| hsa-miR-93-5p | ENSG00000232104 | RFX3-AS1 |
| hsa-miR-93-5p | ENSG00000260912 | AL158206.1 |
| hsa-miR-93-5p | ENSG00000281649 | EBLN3P |
| hsa-miR-93-5p | ENSG00000261094 | AC007066.2 |
| hsa-miR-93-5p | ENSG00000203993 | ARRDC1-AS1 |
| hsa-miR-93-5p | ENSG00000239665 | AL157392.3 |
| hsa-miR-93-5p | ENSG00000185904 | LINC00839 |
| hsa-miR-93-5p | ENSG00000226200 | SGMS1-AS1 |
| hsa-miR-93-5p | ENSG00000223502 | AL731537.1 |
| hsa-miR-93-5p | ENSG00000235931 | LINC01553 |
| hsa-miR-93-5p | ENSG00000233871 | DLG5-AS1 |
| hsa-miR-93-5p | ENSG00000230091 | TMEM254-AS1 |
| hsa-miR-93-5p | ENSG00000223482 | NUTM2A-AS1 |
| hsa-miR-93-5p | ENSG00000261438 | AL157394.1 |
| hsa-miR-93-5p | ENSG00000235823 | OLMALINC |
| hsa-miR-93-5p | ENSG00000269609 | RPARP-AS1 |
| hsa-miR-93-5p | ENSG00000130600 | H19 |
| hsa-miR-93-5p | ENSG00000269821 | KCNQ1OT1 |
| hsa-miR-93-5p | ENSG00000285338 | AC091564.7 |
| hsa-miR-93-5p | ENSG00000281880 | PAUPAR |
| hsa-miR-93-5p | ENSG00000245532 | NEAT1 |
| hsa-miR-93-5p | ENSG00000251562 | MALAT1 |
| hsa-miR-93-5p | ENSG00000258297 | AP001157.1 |
| hsa-miR-93-5p | ENSG00000255507 | AP003031.2 |
| hsa-miR-93-5p | ENSG00000255409 | RSF1-IT1 |
| hsa-miR-93-5p | ENSG00000279900 | AP001767.4 |
| hsa-miR-93-5p | ENSG00000272917 | AC010186.4 |
| hsa-miR-93-5p | ENSG00000284634 | AC092821.3 |
| hsa-miR-93-5p | ENSG00000247498 | GPRC5D-AS1 |
| hsa-miR-93-5p | ENSG00000275097 | AC024940.6 |
| hsa-miR-93-5p | ENSG00000276900 | AC023157.3 |
| hsa-miR-93-5p | ENSG00000257337 | AC068888.1 |
| hsa-miR-93-5p | ENSG00000228630 | HOTAIR |
| hsa-miR-93-5p | ENSG00000276853 | AC026124.2 |
| hsa-miR-93-5p | ENSG00000280088 | AC126474.2 |
| hsa-miR-93-5p | ENSG00000281344 | HELLPAR |
| hsa-miR-93-5p | ENSG00000280120 | AC073857.1 |
| hsa-miR-93-5p | ENSG00000256092 | AC137767.1 |
| hsa-miR-93-5p | ENSG00000278112 | AC145423.3 |
| hsa-miR-93-5p | ENSG00000279466 | AC073911.2 |
| hsa-miR-93-5p | ENSG00000278291 | AL161772.1 |
| hsa-miR-93-5p | ENSG00000176124 | DLEU1 |
| hsa-miR-93-5p | ENSG00000261553 | AL137782.1 |
| hsa-miR-93-5p | ENSG00000259321 | AL136295.2 |
| hsa-miR-93-5p | ENSG00000257621 | PSMA3-AS1 |
| hsa-miR-93-5p | ENSG00000258301 | VASH1-AS1 |
| hsa-miR-93-5p | ENSG00000258593 | AL583810.1 |
| hsa-miR-93-5p | ENSG00000257151 | PWAR6 |
| hsa-miR-93-5p | ENSG00000224078 | SNHG14 |
| hsa-miR-93-5p | ENSG00000259488 | AC023355.1 |
| hsa-miR-93-5p | ENSG00000244879 | GABPB1-AS1 |
| hsa-miR-93-5p | ENSG00000259476 | AC018904.2 |
| hsa-miR-93-5p | ENSG00000259771 | AC092756.1 |
| hsa-miR-93-5p | ENSG00000259248 | USP3-AS1 |
| hsa-miR-93-5p | ENSG00000281183 | NPTN-IT1 |
| hsa-miR-93-5p | ENSG00000260274 | AC068338.2 |
| hsa-miR-93-5p | ENSG00000278600 | AC015871.3 |
| hsa-miR-93-5p | ENSG00000259642 | ST20-AS1 |
| hsa-miR-93-5p | ENSG00000259416 | AC021739.3 |
| hsa-miR-93-5p | ENSG00000259275 | AC087477.2 |
| hsa-miR-93-5p | ENSG00000259583 | AC015712.2 |
| hsa-miR-93-5p | ENSG00000282907 | Z98883.1 |
| hsa-miR-93-5p | ENSG00000263033 | AC007220.1 |
| hsa-miR-93-5p | ENSG00000260280 | SLX1B-SULT1A4 |
| hsa-miR-93-5p | ENSG00000279789 | AC120114.4 |
| hsa-miR-93-5p | ENSG00000213599 | SLX1A-SULT1A3 |
| hsa-miR-93-5p | ENSG00000239791 | AC002310.2 |
| hsa-miR-93-5p | ENSG00000278133 | AC135050.6 |
| hsa-miR-93-5p | ENSG00000278928 | AC136621.1 |
| hsa-miR-93-5p | ENSG00000261519 | AC010542.4 |
| hsa-miR-93-5p | ENSG00000270165 | AC010530.1 |
| hsa-miR-93-5p | ENSG00000259768 | AC004943.2 |
| hsa-miR-93-5p | ENSG00000271009 | AC116667.1 |
| hsa-miR-93-5p | ENSG00000260816 | AC027279.1 |
| hsa-miR-93-5p | ENSG00000261175 | LINC02188 |
| hsa-miR-93-5p | ENSG00000279432 | AC015799.1 |
| hsa-miR-93-5p | ENSG00000262251 | AC087388.1 |
| hsa-miR-93-5p | ENSG00000277511 | AC116407.2 |
| hsa-miR-93-5p | ENSG00000265139 | AC005899.3 |
| hsa-miR-93-5p | ENSG00000278954 | AC130686.1 |
| hsa-miR-93-5p | ENSG00000244649 | LINC02086 |
| hsa-miR-93-5p | ENSG00000279089 | AC005839.1 |
| hsa-miR-93-5p | ENSG00000279281 | AC015883.1 |
| hsa-miR-93-5p | ENSG00000224738 | AC099850.1 |
| hsa-miR-93-5p | ENSG00000267416 | AC025048.4 |
| hsa-miR-93-5p | ENSG00000215769 | ARHGAP27P1-BPTFP1-KPNA2P3 |
| hsa-miR-93-5p | ENSG00000279573 | AC134407.2 |
| hsa-miR-93-5p | ENSG00000163597 | SNHG16 |
| hsa-miR-93-5p | ENSG00000234912 | SNHG20 |
| hsa-miR-93-5p | ENSG00000279187 | AC027601.5 |
| hsa-miR-93-5p | ENSG00000279066 | HEXDC-IT1 |
| hsa-miR-93-5p | ENSG00000262652 | AC124283.3 |
| hsa-miR-93-5p | ENSG00000263884 | AP000845.1 |
| hsa-miR-93-5p | ENSG00000266805 | AP005432.1 |
| hsa-miR-93-5p | ENSG00000268573 | AC011815.1 |
| hsa-miR-93-5p | ENSG00000260578 | AC110597.1 |
| hsa-miR-93-5p | ENSG00000267287 | AC068473.3 |
| hsa-miR-93-5p | ENSG00000279203 | AC005785.2 |
| hsa-miR-93-5p | ENSG00000279425 | AC092279.2 |
| hsa-miR-93-5p | ENSG00000280106 | AC008555.8 |
| hsa-miR-93-5p | ENSG00000267254 | AC020928.1 |
| hsa-miR-93-5p | ENSG00000267152 | AC093227.1 |
| hsa-miR-93-5p | ENSG00000279539 | AC006486.2 |
| hsa-miR-93-5p | ENSG00000186019 | AC021092.1 |
| hsa-miR-93-5p | ENSG00000279095 | AC243964.3 |
| hsa-miR-93-5p | ENSG00000260160 | AC011468.1 |
| hsa-miR-93-5p | ENSG00000269825 | AC022150.4 |
| hsa-miR-93-5p | ENSG00000267265 | AC011476.3 |
| hsa-miR-93-5p | ENSG00000276570 | AC010327.5 |
| hsa-miR-93-5p | ENSG00000268205 | AC005261.1 |
| hsa-miR-93-5p | ENSG00000176593 | AC008969.1 |
| hsa-miR-93-5p | ENSG00000228293 | AL049712.1 |
| hsa-miR-93-5p | ENSG00000277425 | AL121890.4 |
| hsa-miR-93-5p | ENSG00000277938 | AL035252.3 |
| hsa-miR-93-5p | ENSG00000277692 | AL121583.1 |
| hsa-miR-93-5p | ENSG00000260032 | NORAD |
| hsa-miR-93-5p | ENSG00000223891 | OSER1-AS1 |
| hsa-miR-93-5p | ENSG00000227477 | STK4-AS1 |
| hsa-miR-93-5p | ENSG00000280594 | BTG3-AS1 |
| hsa-miR-93-5p | ENSG00000261610 | AP000265.1 |
| hsa-miR-93-5p | ENSG00000280604 | AJ239328.1 |
| hsa-miR-93-5p | ENSG00000093100 | AC016026.1 |
| hsa-miR-93-5p | ENSG00000230513 | THAP7-AS1 |
| hsa-miR-93-5p | ENSG00000278657 | AC245452.5 |
| hsa-miR-93-5p | ENSG00000279217 | Z95114.1 |
| hsa-miR-93-5p | ENSG00000279738 | AL022311.1 |
| hsa-miR-93-5p | ENSG00000280434 | AL031595.3 |
| hsa-miR-93-5p | ENSG00000280011 | AL031595.2 |
| hsa-miR-93-5p | ENSG00000197182 | MIRLET7BHG |
| hsa-miR-93-5p | ENSG00000260822 | AC004656.1 |
| hsa-miR-93-5p | ENSG00000241886 | AC112496.1 |
| hsa-miR-93-5p | ENSG00000226310 | AL022157.1 |
| hsa-miR-93-5p | ENSG00000229807 | XIST |
| hsa-miR-93-5p | ENSG00000280195 | AC245140.2 |
| hsa-miR-93-5p | ENSG00000233864 | TTTY15 |

**Supplementary Table 2** LncRNAs that may affect the expression of the three miRNAs were obtained through the online website.

| Gene | primers sequences (strand) |
| --- | --- |
| β-actin | Forward: 5’-ATAGCACAGCCTGGATAGCAACGTAC-3’ |
|  | Reverse: 5’- CACCTTCTACAATGAGCTGCGTGTG-3’ |
| LINC01798 | Forward: 5’- TTGAGCCAAACAAAGGGGGT-3’ |
|  | Reverse: 5’- GACAGCAAAATGCTCCCGTG-3’ |
| miR-17-5p | Forward: 5’- TGCTTACAGTGCAGGTAG-3’ |
|  | Reverse: 5’- GAACATGTCTGCGTATCTC-3’ |
| ITGA8 | Forward: 5’- AGTTACCTTGAATTGGTTGCTGG-3’ |
|  | Reverse: 5’- ATGCCATCTGTTCTCCCGTG -3’ |
| U6 | Forward: 5’- CTCGCTTCGGCAGCACAT-3’ |
|  | Reverse: 5’- TTTGCGTGTCATCCTTGCG-3’ |
| SELP | Forward: 5’- GTCCTTAAGGTTTCCATAAACACCC-3’ |
|  | Reverse: 5’- GGAAACAGGGTTGGTCCAGA-3’ |
| EDNRB | Forward: 5’-CTGGCCATTTGGAGCTGAGA-3’ |
|  | Reverse: 5’- AGCAACAGCTCGATATCTGTCA-3’ |
| CD40LG | Forward: 5’-GCGGCACATGTCATAAGTGAGG-3’ |
|  | Reverse: 5’- GTCCTTGTCTTTTAACGGTCAGC-3’ |
| C10orf54 | Forward: 5’-GGCAAAGATGCACCATCCAA-3’ |
|  | Reverse: 5’-CTGCAGCCGTGATGTTTTCAC-3’ |
| has-miR-17-5p-RT | GTGCAGGGTCCGAGGTCAGAGCCACCTGGGC  AATTTTTTTTTTTCTACCT |
| si-LINC01798 | GCAGTGTCCAGGAATTAGA |
| miR-17-5p inhibitor | CUACCUGCACUGUAAGCACUUUG |

**Supplementary Table 3** Primer sequences of the target genes.
