# Supplementary material for: LINC01798/miR-17-5p axis regulates ITGA8 and causes changes in tumor microenvironment and stemness in lung adenocarcinoma
Source: Front Immunol. 2023 Feb 23;14:1096818. doi: 10.3389/fimmu.2023.1096818 (PMC9995370; doi:10.3389/fimmu.2023.1096818)
Supplement: Supplementary file 2 [file DataSheet_2.docx]

<https://www.jianguoyun.com/c/sd/165c912/1acdf4271f352003>
